# Workplace interventions for chronic musculoskeletal disorders: a systematic review

**DOI:** 10.1136/bmjopen-2025-115276

**Published:** 2026-06-22

**Authors:** Glykeria Skamagki, Colin J Greaves, Ruby M Shrestha, Afroditi Stathi

**Affiliations:** 1 University of Birmingham School of Sport Exercise and Rehabilitation Sciences, Birmingham, UK; 2 School of Geography, Earth and Environmental Sciences, University of Birmingham, Birmingham, UK

**Keywords:** Systematic Review, Workplace, Musculoskeletal disorders, Chronic Disease, Disease Management

## Abstract

**Abstract:**

**Objectives:**

To provide an updated systematic review of the effectiveness of workplace interventions for chronic musculoskeletal disorders, identify key intervention components and assess their impact on health and work-related outcomes.

**Design:**

Systematic review of randomised controlled trials.

**Data sources:**

MEDLINE, EMBASE, CINAHL, PsycINFO, Scopus and Web of Science were searched for studies published between 2018 and 2024. Grey literature sources were also consulted.

**Eligibility criteria:**

Randomised controlled trials and cluster-randomised trials evaluating workplace interventions targeting adults with chronic musculoskeletal disorders were included. Outcomes of interest included pain, functional outcomes, quality of life and work-related outcomes (eg, work ability, productivity, presenteeism and absenteeism).

**Data extraction and synthesis:**

Two reviewers independently screened studies, extracted data and assessed risk of bias using the Cochrane Risk of Bias 2 tool. Due to heterogeneity, only a narrative synthesis was conducted.

**Results:**

23 studies involving 2456 participants were included and classified into physical exercise, ergonomic and multicomponent interventions. Physical exercise interventions consistently improved pain and functional outcomes. Digital delivery of exercise interventions demonstrated comparable effectiveness to face-to-face or workplace-based approaches. Multicomponent interventions, combining exercise with ergonomic strategies, demonstrated the most consistent benefits across outcomes. Evidence for work-related outcomes was limited and inconsistent, and quality of life was assessed in only a small number of studies.

**Conclusions:**

Workplace interventions, particularly multicomponent approaches combining exercise, ergonomics and education, can be effective in improving health outcomes in chronic musculoskeletal disorders. However, evidence on work-related outcomes remains limited and inconsistent.

**PROSPERO registration number:**

CRD420261289218.

**STRENGTHS AND LIMITATIONS OF THIS STUDY**

- This review followed Preferred Reporting Items for Systematic reviews and Meta-Analyses (PRISMA) guidelines and used a comprehensive search strategy across multiple databases and grey literature sources.
- Two reviewers independently screened studies, extracted data and assessed risk of bias to reduce selection and extraction bias.
- Only randomised controlled trials (RCTs) and cluster RCTs were included, strengthening the methodological quality of the evidence base.
- Considerable heterogeneity in intervention types, outcome measures and follow-up periods prevented meta-analysis and limited comparability across studies.
- Work-related outcomes such as absenteeism, presenteeism and work ability were inconsistently reported across studies.

## Introduction

 Chronic musculoskeletal disorders (CMSDs) are a major occupational health challenge due to their high prevalence, impact on physical functionality, quality of life and their role in absenteeism and presenteeism.^1^ These conditions impose significant economic burdens through healthcare costs and lost productivity, adding social strain on workers and their families. Around one-third of the UK population (20.3 million people) live with a musculoskeletal condition^2^ experiencing chronic pain and productivity loss. A healthy work environment enhances employee well-being and organisational outcomes by increasing productivity and reducing absenteeism and presenteeism.^3 4^

The COVID-19 pandemic has significantly impacted working conditions, leading to an increase in CMSDs and mental health conditions.^5^ Recent studies have highlighted the correlation between increased sedentary behaviour during remote work and the rise in musculoskeletal pain, particularly in the upper back, neck and shoulders.^6^ The rapid shift to remote work has introduced new health challenges, including inadequate ergonomic set-ups and prolonged sedentary behaviour, which have exacerbated musculoskeletal issues among employees.^7^ In physically demanding jobs the pandemic has led to increased physical and psychosocial burdens, raising the risk of work-related musculoskeletal disorders.^8^ Increased job demands and insufficient recovery time have further strained workers’ musculoskeletal health.

Before the pandemic, research emphasised the importance of workplace intervention strategies for managing CMSDs.^9 10^ However, these studies often had limitations like small sample sizes, short follow-up periods and a narrow focus. They also did not fully consider diverse work environments or the challenges of remote and hybrid work models. The evolving work landscape has made updated research on CMSDs management urgent. Recent studies explore a wider range of interventions, including digital health solutions and comprehensive wellness programmes, offering critical insights for effective CMSDs management in the flexible, post-pandemic workplace.

This systematic review provides an updated synthesis of workplace interventions for CMSDs, incorporating recent evidence and addressing limitations of previous reviews, particularly in the context of evolving work environments including remote and hybrid work. It reassesses the effectiveness of workplace management strategies for CMSDs and identifies best practices for both traditional and remote work environments, aiming to improve health outcomes and productivity.

## Methods

### Search strategy

This systematic review was conducted following the methodologies outlined in the Cochrane Handbook for Systematic Reviews of Interventions.^11^ The Population, Intervention, Comparison, Outcome framework was employed to structure the research question (table 1) and guide the identification of relevant inclusion and exclusion criteria (table 2). This structured approach ensured a comprehensive and systematic search for studies that evaluated the effectiveness of workplace interventions for managing CMSDs.

**Table 1:** PICO approach

Population/problem	Employees with CMSDs
Intervention	Workplace strategies/interventions to manage CMSDs
Comparison	Control condition (usual care, minimal intervention or alternative intervention)
Outcome	Pain, functional outcomes, quality of life and work-related outcomes (work ability, productivity, presenteeism and absenteeism)

CMSDs, chronic musculoskeletal disorders.

**Table 2:** Inclusion and exclusion criteria

Participant inclusion criteria	Participant exclusion criteria
Working age male and female adults (18–68 years)	Specific pathological conditions (eg, tumours, infections, fractures)
All sectors and types of jobs	Hypertension or cardiovascular diseases, symptomatic disc prolapses or severe disorders of the cervical spine, postoperative conditions in the neck and shoulder region, history of severe trauma and pregnancy.
Workers with reported CMSDs (12 weeks or more) at any area of the body	Acute musculoskeletal disorders
Group-based and individual interventions conducted at the workplace	Guidelines, policies, recommendations
Interventions focused on management of CMSDs	Interventions focused on prevention and return to work
RCT studies or cRCTs	Surveys and qualitative studies

CMSDs, chronic musculoskeletal disorders; cRCT, cluster RCT; RCT, randomised controlled trial.

The literature search focused on studies published between 2018 and 2024. Databases searched included MEDLINE, CINAHL, AMED, Cochrane, PsycINFO, Academic Search Complete and PEDro. Additionally, Scopus was used for post-publication citation searching to identify any further relevant articles. The search strategy incorporated Boolean operators (AND/OR/NOT), Subject Headings, alternative spellings, acronyms and wildcards to maximise the retrieval of relevant studies. A limited search of grey literature was also conducted, targeting relevant websites such as the Institute for Work and Health, the Institution of Occupational Safety and Health and the European Agency for Safety and Health at Work. This step ensured that any non-indexed but relevant studies or reports were not overlooked. An example of the search strategy can be found in (online supplemental file 1).

### Selection of studies

The search strategy identified 2148 studies, which were managed and organised using Covidence software. Covidence was instrumental in efficiently handling the removal of duplicate records and streamlining the entire screening process. During the first stage of screening, the titles and abstracts of all studies were independently reviewed by two reviewers (GS and RS) to assess their relevance against the predetermined inclusion criteria. Studies that appeared to meet the criteria or where eligibility was unclear underwent a full-text review to confirm their inclusion in the systematic review. Disagreements between the reviewers were resolved through discussion, or by consulting a third reviewer (AS) when necessary.

### Eligibility criteria

#### Inclusion criteria

Randomised controlled trial studies (RCTs) and cluster RCT studies (cRCTs) were included if they tested workplace interventions for managing CMSDs (conditions lasting 12 weeks or more). Participants had to be adults over 18 years old, including both males and females. Intervention studies were included only if they focused on the management of CMSDs.

#### Exclusion criteria

Studies were excluded if they focused only on prevention or return-to-work strategies, involved participants with acute musculoskeletal or other chronic conditions or were conducted in non-workplace settings like hospitals or private rehabilitation centres. Guidelines, policies and non-research-based recommendations were also excluded to focus on empirical evidence. A summary table of inclusion and exclusion criteria can be found in table 2.

### Data collection

After selecting the studies, data extraction was done using a standardised form in Covidence. Collected data included study design, country, participant characteristics, intervention details, outcome measures and main results. Two reviewers (GS and RS) independently performed the extraction to minimise bias, resolving any disagreements collaboratively.

### Risk of bias assessment

The risk of bias within the included studies was rigorously assessed using the Cochrane Risk of Bias 2 (RoB 2) tool which is widely recognised for its comprehensive approach to evaluating potential biases in RCTs. Each study was carefully evaluated across these domains and classified as having a low risk of bias, some concerns or a high risk of bias. The overall quality of the included studies was determined by combining the assessments from each domain. Studies with a low risk of bias in all domains were considered high quality, while those with one or more domains rated as high risk were considered to have potential limitations in their findings. Risk of bias was assessed using the RoB 2 tool and summarised using a traffic-light visualisation.

### Data synthesis

Due to heterogeneity in intervention types, outcome measures and follow-up periods, a meta-analysis was not conducted. A narrative synthesis approach was undertaken in accordance with established guidance for systematic reviews. Studies were grouped according to intervention type (exercise, ergonomic and multicomponent interventions), and findings were synthesised by examining the direction, consistency and magnitude of effects across studies. Outcomes were considered in relation to both clinical (pain, functional outcomes, quality of life) and work-related domains (work ability, productivity, presenteeism and absenteeism) to identify patterns in effectiveness across intervention types.

### Outcomes

The outcomes of interest were categorised into clinical outcomes (pain, functional outcomes and quality of life) and work-related outcomes (work ability, productivity, presenteeism and absenteeism). Outcomes were primarily assessed using self-reported measures, with objective measures included where available.

## Results

### Selection of studies

**Figure 1:**
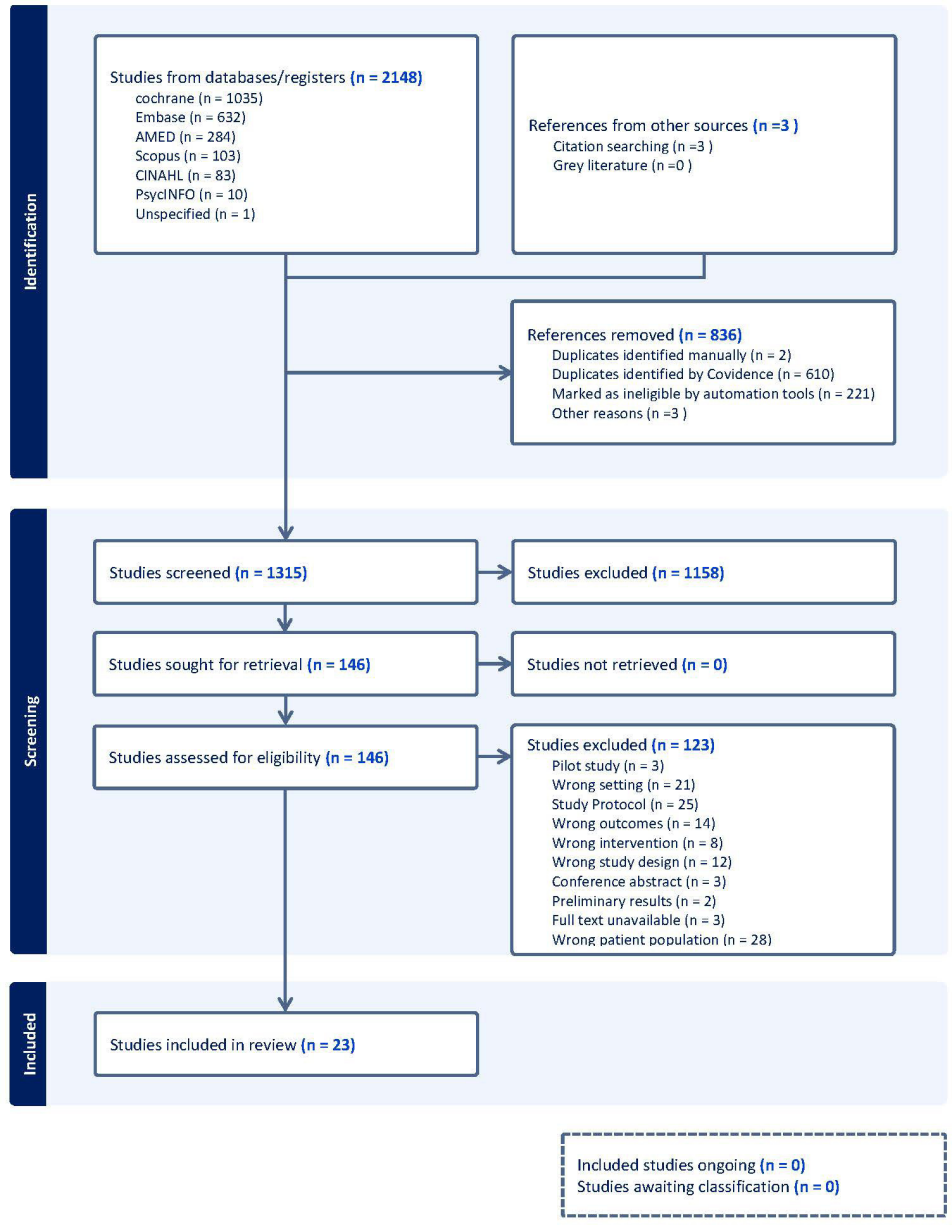
Preferred Reporting Items for Systematic reviews and Meta-Analyses (PRISMA).

This systematic review included 23 studies published between 2018 and 2024, each evaluating interventions aimed at managing CMSDs within various occupational settings (figure 1). The selected studies reflect a broad spectrum of research efforts, encompassing diverse geographical locations, occupational groups, intervention strategies and outcome measures. The studies were conducted across 12 different countries, illustrating the global relevance of managing CMSDs in the workplace.

### Study characteristics

The study designs included RCTs, with 19 out of 23 studies using this method. Four studies used cRCTs, randomising groups rather than individuals, which is relevant for workplace settings. Sample sizes ranged from 27 to 250 participants, with larger studies providing more robust data for generalising conclusions about intervention effectiveness.

The mean age of participants varied across studies, reflecting the diversity of the working population. For example, Bernardelli *et al*^12^ focused on an older population with a mean age of 51.1 years, typical of healthcare workers, who often experience chronic low back pain due to the physical demands of their job. In contrast, Shariat *et al*^13^ included a younger cohort with a mean age of 29.5 years, reflecting the office worker population, which faces different ergonomic challenges.

Occupational settings across the studies were diverse, including office workers,^14 15^ healthcare professionals,^16 17^ industrial workers,^18^ military personnel^19^ and manual labourers.^20^ This diversity allowed for an examination of how different job demands and work environments influence the effectiveness of interventions.

### Types of interventions

Interventions were categorised based on their primary content into three main groups:

1. Exercise-based interventions, including strength training, stretching, neuromuscular exercises and functional rehabilitation programmes.
2. Ergonomic interventions, including workstation modifications and adjustments to work tasks or environments.
3. Multicomponent interventions, combining physical, educational, behavioural or psychosocial elements.

Interventions were delivered through different modes, including face-to-face (individual or group-based) and digital platforms (eg, software-based or virtual interventions).

### Outcome measures

Across the included studies, pain outcomes were assessed in all 23 studies, while functional outcomes were reported in the majority of studies. Work-related outcomes were assessed in a limited number of studies, with work ability reported in a small number of trials, productivity assessed in only a few studies and presenteeism reported in one study. Absenteeism was not consistently measured across studies. Quality of life was assessed in a small number of studies, primarily within multicomponent interventions. Exercise-based interventions were the most frequently evaluated (n=13), followed by multicomponent interventions (n=9), with only one study assessing a purely ergonomic intervention (n=1).

Pain was universally measured, typically using Visual Analogue Scale or Numerical Pain Rating Scale. Functional outcomes were commonly evaluated using the Oswestry Disability Index and the Roland-Morris Disability Questionnaire, both of which are well-established tools for assessing the impact of CMSDs on daily activities. Work-related outcomes were assessed in a limited number of studies. Quality of life was assessed using instruments such as the 36-Item Short Form Survey and the EuroQol Group (version EQ-5D).

### Quality appraisal

**Figure 2:**
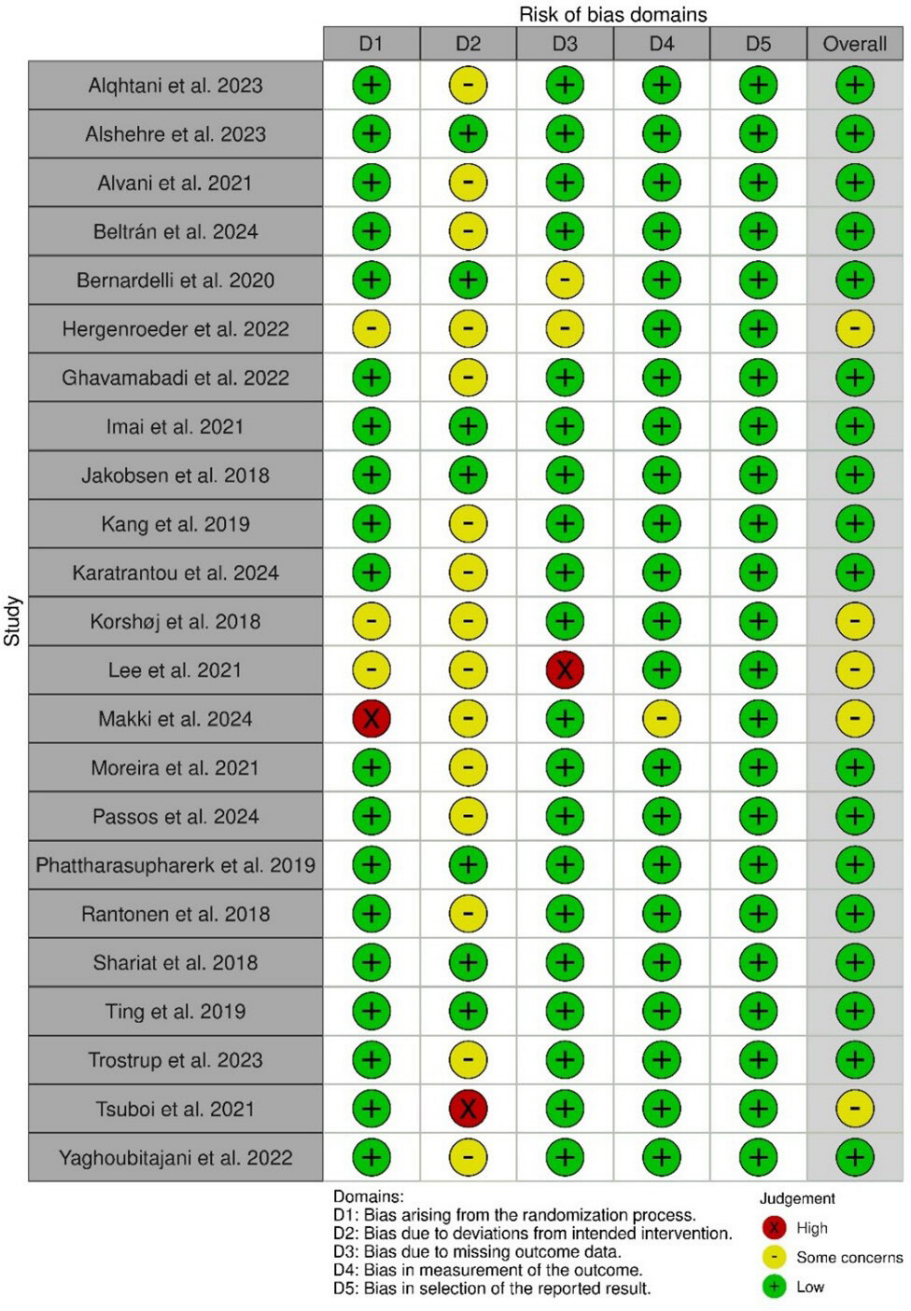
Visualising risk-of-bias assessments with ‘traffic lights’.

A critical appraisal of the 23 studies included in this systematic review was conducted to assess the quality and reliability of the evidence presented (figure 2). This appraisal focused on evaluating the risk of bias, methodological rigour and the overall validity of the studies’ findings, using the RoB 2 tool. The overall quality of the included studies demonstrated a low risk of bias across the assessed domains, indicating a high level of methodological rigour. These studies generally offered robust evidence supporting the effectiveness of workplace interventions in managing CMSDs. Nevertheless, certain studies exhibited methodological limitations, particularly in the areas of blinding and handling of incomplete data, which should be considered when interpreting their findings.

Among the studies assessed, some stood out for their high quality. For example, two studies were notable for their rigorous design and low risk of bias across all domains.^12 18^ These studies implemented robust randomisation procedures, maintained strict adherence to intervention protocols and provided comprehensive reporting, making their findings highly reliable and valuable to the field. Conversely, a few studies exhibited higher risks of bias, particularly in areas related to randomisation and outcome measurement.^21 22^ These studies often lacked sufficient detail on how randomisation was conducted or relied heavily on self-reported outcomes without blinding, which could introduce significant bias.

### Summary of effectiveness data

This section provides a narrative synthesis of the effectiveness of workplace interventions for managing CMSDs, structured according to the predefined intervention categories. Of the 23 included studies, the majority evaluated exercise-based interventions (n=13), followed by multicomponent interventions (n=9), with only one (n=1) study examining a purely ergonomic intervention. The effectiveness of workplace interventions is presented according to intervention type. A summary of study characteristics is presented in table 3 and full details are presented in online supplemental file 2.

**Table 3:** Summary of included RCT and cRCT studies by intervention type, sample size and outcome measures

Exercise-based interventions		
Author	Sample size (baseline and follow-up)	Outcomes (measurement instruments)
Alqhtani *et al*^23^	Baseline: IG=30, CG=30 Follow-up: IG=30, CG=30	Pain, functional disability (VAS, ODI)
Alvani *et al*^19^	Baseline: IG=15, CG=15 Follow-up: IG=15, CG=15	Pain, functional disability (VAS, ODI)
Beltrán *et al*^20^	Baseline: IG=22, CG=22 Follow-up: n=42	Pain, shoulder function (NPRS, SPADI)
Bernardelli *et al*^12^	Baseline: IG=47, CG=37 Follow-up: n=84	Pain, functional disability (VAS, RMDQ)
Ibrahimi Ghavamabadi *et al*^18^	Baseline: IG=125, CG=125 Follow-up: IG=125, CG=125	Pain, functional disability (VAS, ODI)
Jakobsen *et al*^17^	Baseline: IG=111, CG=89 Follow-up: n=184	Pain, work ability (VAS, Work Ability Index)
Kang *et al*^24^	Baseline: IG=15, CG=15 Follow-up: n=29	Pain, hand function (VAS, AUSCAN)
Karatrantou and Gerodimos^25^	Baseline: IG=35, CG=35 Follow-up: n=70	Pain, functional disability (VAS, ODI)
Korshøj *et al*^41^	Baseline: IG=57, CG=59 Follow-up: IG=57, CG=59	Pain, work ability (VAS, Work Ability Index)
Moreira *et al*^27^	Baseline: IG=46, CG=44 Follow-up: IG=46, CG=44	Pain, quality of life (VAS, SF-36)
Passos *et al*^42^	Baseline: IG=22, CG=22 Follow-up: n=44	Pain, functional disability (NPRS, ODI)
Phattharasupharerk *et al*^43^	Baseline: IG=36, CG=36 Follow-up: n=65	Pain, functional disability (VAS, ODI)
Makki *et al*^22^	Baseline: IG=33, CG=33 Follow-up: n=66	Pain, posture, functional outcomes (VAS, posture analysis tools)
**Ergonomic interventions**		
Author	Sample size (baseline and follow-up)	Outcomes (measurement instruments)
Lee *et al*^15^	Baseline: IG=32, CG=32 Follow-up: n=64	Pain, musculoskeletal symptoms (VAS, Nordic Musculoskeletal Questionnaire)
Yaghoubitajani *et al*^26^	Baseline: IG1=12, IG2=12, CG=12 Follow-up: IG1=12, IG2=12, CG=12	Posture, musculoskeletal alignment (postural assessment tools)
**Multicomponent interventions**		
Author	Sample size (baseline and follow-up)	Outcomes (measurement instruments)
Alshehre *et al*^14^	Baseline: IG=12, CG=12 Follow-up: n=24	Pain, disability, range of motion (VAS, Neck Disability Index)
Hergenroeder *et al*^44^	Baseline: IG=12, CG=12 Follow-up: n=24	Pain, physical activity, sedentary behaviour (VAS, activity monitoring tools)
Imai *et al*^16^	Baseline: IG=51, CG=53 Follow-up: IG=51, CG=53	Pain, work productivity, quality of life (VAS, Work Productivity and Activity Impairment Questionnaire)
Rantonen *et al*^28^	Baseline: IG1=43, IG2=43, IG3=40, CG=50 Follow-up: same as baseline	Pain, work ability (VAS, disability not clearly specified)
Shariat *et al*^13^	Baseline: IG1=43, IG2=37, IG3=34, CG=28 Follow-up: n=142	Pain, posture, musculoskeletal symptoms (VAS, posture assessment tools)
Ting *et al*^29^	Baseline: IG1=52, IG2=45 Follow-up: not reported	Pain, functional disability (VAS, Neck Disability Index)
Trøstrup *et al*^30^	Baseline: IG=57, CG=52 Follow-up: n=93	Pain, work ability, function (VAS, DASH)
Tsuboi *et al*^21^	Baseline: IG=16, CG=13 Follow-up: IG=16, CG=13	Pain, functional disability (VAS, ODI)

AUSCAN, Australian/Canadian Osteoarthritis Hand Index; CG, control group; DASH, Disabilities of the Arm, Shoulder and Hand; IG, intervention group; NPRS, Numerical Pain Rating Scale; ODI, Oswestry Disability Index; RMDQ, Roland-Morris Disability Questionnaire; SF-36, 36-Item Short Form Survey; SPADI, Shoulder Pain and Disability Index; VAS, Visual Analogue Scale.

### Exercise-based interventions

Thirteen studies evaluated exercise-based interventions, including strength training, stretching, neuromuscular exercise, movement-based approaches and functional rehabilitation programmes. Across these studies, consistent improvements in pain outcomes were observed. For example, Alqhtani *et al*^23^ reported reductions in pain at 6 weeks (p<0.001), with significant between-group differences favouring the intervention (p<0.05). Similarly, Ibrahimi Ghavamabadi *et al*^18^ reported significant reductions in pain (p<0.001) and disability (p<0.001), with consistent between-group differences (p<0.001). Jakobsen *et al*^17^ demonstrated reductions in pain across multiple body regions (eg, lower back p<0.001), favouring workplace-based exercise compared with home-based programmes.

Similarly, Kang *et al*^24^ demonstrated improvements in hand function (p<0.001) and grip strength (p<0.001), with significant between-group differences (p=0.015). Longer-term improvements were observed in Karatrantou and Gerodimos^25^ with reductions in pain across all body regions (p<0.001) and improvements in strength (p<0.001). In addition, variability in intervention duration, frequency and supervision limited comparability across studies. Finally, two studies delivered exercise interventions through digital platforms.^22 26^ These reported comparable reductions in pain and functional improvements to face-to-face interventions, although no additional benefit in work-related outcomes was observed.

Overall, evidence for work-related outcomes was limited and inconsistent. Quality of life was assessed in one exercise-based study,^27^ which reported improvements following intervention, although this outcome was not widely evaluated across exercise interventions. Similarly work ability was the most frequently assessed occupational outcome, while productivity and presenteeism were rarely measured and absenteeism was not consistently reported. For example, one of the studies reported a small but statistically significant improvement in physical workability (p=0.048), favouring the online-supervised intervention, although no significant differences were observed for sick leave.^26^

### Ergonomic interventions

Only one study evaluated a purely ergonomic intervention in isolation. Lee *et al*^15^ reported reductions in pain across several body regions, with significant between-group differences observed for neck (p<0.01), shoulder (p=0.02), upper back (p=0.03) and wrist/hand (p<0.01). However, no significant differences were observed for lower back (p=0.19), hip (p=0.19), knee (p=0.64) or foot/ankle pain (p=0.43). Effect sizes were not reported. In contrast, studies incorporating ergonomic components within broader intervention programmes reported more favourable results, suggesting that ergonomic modifications may be more effective when combined with other approaches (section below).

### Multicomponent interventions

Multicomponent interventions, combining physical, ergonomic, educational and behavioural elements, demonstrated improvements in both clinical outcomes and, in some cases, work-related outcomes. Quality of life outcomes were also reported in a small number of studies within this category. For example, two studies reported significant improvements in QoL measures, although this outcome was not consistently assessed across all multicomponent interventions.^14 16^ In addition, multicomponent interventions demonstrated improvements across both clinical and, in some cases, work-related outcomes. Alshehre *et al*^14^ reported reductions in pain (p<0.001), disability (p<0.001) and improvements in quality of life (p<0.001), with consistent between-group differences (p<0.001). Similarly, Imai *et al*^16^ demonstrated improvements in pain (p=0.001) and presenteeism (p=0.001), with moderate to large effect sizes (d=0.54–0.94). Rantonen *et al*^28^ reported reductions in disability (p<0.001, d=0.7) and pain (p<0.001, d=0.6), with significant between-group differences.

However, findings were not consistent across all multicomponent interventions. Ting *et al*^29^ reported improvements in work ability at 12 weeks (p=0.03), but not at 12 months (p=0.06) and no significant between-group differences were observed. Similarly, Trøstrup *et al*^30^ found no significant differences between groups for pain or function despite improvements within both groups. Tsuboi *et al*^21^ reported no significant improvements in pain (p=0.965), disability (p=0.085) or productivity (p=0.218), highlighting variability in effectiveness across multicomponent approaches. Overall, multicomponent interventions demonstrated broader effects across outcomes compared with single-component interventions, although results varied depending on intervention design and outcome measures.

## Discussion

This systematic review provides an updated synthesis of workplace interventions for CMSDs and reveals a consistent but important pattern across the evidence base: while pain and functional outcomes were consistently assessed and improved across studies, work-related outcomes were infrequently measured and showed limited and inconsistent findings, restricting conclusions about the impact of interventions on occupational functioning. This distinction highlights a gap between clinical improvement and meaningful occupational recovery.^1^ Reducing symptoms does not necessarily translate into improved work participation, productivity or sustained employability especially if interventions target individual-level impairments without addressing workplace-level barriers, such as job demands, organisational constraints and employer support. Therefore there is a disconnect between the mechanisms targeted by current interventions and the broader determinants of work ability, which include organisational, psychosocial and behavioural factors beyond physical health.

Exercise-based interventions were the most frequently studied and demonstrated consistent benefits across pain and functional outcomes. These findings reinforce the role of exercise in CMSDs management and align with broader evidence highlighting its effectiveness in improving musculoskeletal health.^9 31^ However, the limited translation of these benefits into work-related outcomes suggests that improvements in physical capacity alone may be insufficient to influence work functioning. This may reflect the complex and multifactorial nature of work ability, which is shaped not only by physical health but also by psychosocial factors, workplace demands, organisational culture and job control.^32^ Therefore, interventions that focus solely on physical rehabilitation may not address the broader determinants of work participation.

Although not consistently assessed across studies, adherence may influence the effectiveness of exercise-based interventions, particularly when comparing supervised and unsupervised programmes. Evidence from previous research suggests that adherence to unsupervised exercise interventions tends to decline over time, even when supported through digital platforms^33^ while supervised programmes are associated with improved adherence and technique.^34^ Follow-up periods varied considerably across studies, ranging from approximately 4 weeks to 24 months, which limits conclusions about the sustainability of intervention effects. This indicates uncertainty around the long-term maintenance of clinical improvements following workplace interventions. Future research should explore how different delivery formats, including hybrid approaches, influence adherence and long-term outcomes.

Furthermore, the mode of delivery, including digital and remote interventions, may influence how interventions are implemented rather than their effectiveness alone. In the present review, digital interventions demonstrated comparable clinical outcomes to traditional face-to-face approaches, suggesting they may be suitable for wider implementation, particularly in remote or hybrid work environments.^35^ However, evidence on user engagement, accessibility and implementation was not consistently assessed across studies. This highlights that effectiveness is not determined by delivery mode alone, and that the design and integration of interventions within workplace contexts may play an important role.

The limited integration of psychological support and workplace-specific guidance within digital tools highlights important gaps in how these interventions can be applied in workplace settings. These findings suggest that digital interventions should be viewed as complementary rather than standalone solutions. Their effectiveness is likely to depend on how they are integrated within broader intervention strategies and supported by mechanisms that promote sustained engagement.

The findings of this review also highlight the limited role of ergonomic interventions when implemented in isolation. This aligns with recent systematic reviews indicating that ergonomic interventions can reduce musculoskeletal symptoms, but with variable and context-dependent effects.^31^ For example, Santos *et al*^36^ reported statistically significant reductions in musculoskeletal pain across several body regions, including the lower back, neck and wrists, although effects were not consistent across all outcomes and heterogeneity across studies was moderate to high. This suggests that while ergonomic interventions can contribute to symptom reduction, their effectiveness is context-dependent and may be insufficient to address the complexity of chronic musculoskeletal conditions. Importantly, ergonomic interventions primarily target exposure to physical risk factors, such as posture, load and repetitive movements and may therefore be more closely aligned with prevention rather than the management of established chronic conditions.

Multicomponent interventions demonstrated the most consistent and comprehensive pattern of effectiveness across outcomes in this review. Programmes combining exercise, ergonomic adjustments and behavioural or educational components were more likely to improve clinical outcomes and, in some cases, work-related outcomes, compared with single-component approaches. Addressing both individual and workplace-level factors may be necessary to achieve broader and more sustained effects. These findings align with previous research demonstrating that multidisciplinary and integrated interventions are more effective in improving return-to-work outcomes than single-component approaches, particularly when workplace and clinical components are combined.^37 38^

The effectiveness of these interventions likely reflects their ability to address multiple interacting determinants of CMSDs, including physical capacity, workplace exposure and, in some cases, behavioural or educational components. Therefore, there is a need to move from symptom-focused approaches towards interventions that consider the broader context of work participation. Within this framework, ergonomic components may function as an enabling element by reducing ongoing physical strain and supporting sustained participation in work tasks, rather than acting as the primary driver of clinical improvement. This is consistent with previous evidence suggesting that combining ergonomic adjustments with physical activity may be more cost-effective in reducing musculoskeletal discomfort and associated workplace burden.^39^ Overall, interventions targeting a single domain may be insufficient to achieve meaningful improvements in work-related outcomes, reinforcing the need for integrated approaches.

A particularly important finding is the limited and inconsistent use of work-related outcomes such as absenteeism and presenteeism across studies. While pain was assessed in all included studies, work-related outcomes were reported less frequently, indicating that workplace interventions for CMSDs continue to be evaluated primarily in clinical rather than occupational terms. Absence-based measures alone may not capture the full impact of interventions. Evidence suggests that productivity losses related to presenteeism may exceed those associated with absenteeism, yet presenteeism remains difficult to measure and is often under-represented in the literature.^40^ Furthermore, variation in how work-related outcomes are defined and measured limits comparability across studies and weakens interpretation of effectiveness.^35^ Therefore, there is a need for more consistent inclusion of standardised work-related measures such as absenteeism, presenteeism and work ability in future research.

From a practical and policy perspective, these findings support the development of integrated and context-sensitive workplace health strategies. Interventions should combine physical exercise, ergonomic adjustments and behavioural or educational components, while being tailored to specific occupational contexts and workforce needs. There is a need to move beyond symptom-focused approaches towards strategies that address work participation and sustainability. This is particularly relevant in the context of ageing workforces and the increasing prevalence of chronic conditions, where long-term management and retention in work are key priorities. Overall, this review confirms that although effective strategies for improving clinical outcomes are well established, translating these improvements into meaningful workplace outcomes remains a key challenge.

## Limitations

This review should be considered in light of the methodological heterogeneity and variable risk of bias across included studies, which may influence the consistency and reliability of reported effects. The heterogeneity of studies, including variation in intervention types, outcome measures and follow-up periods, prevented a meta-analysis. Inconsistent reporting of effect sizes and adherence metrics further complicates the evaluation of practical significance. The reliance on narrative synthesis, due to heterogeneity, may limit the ability to quantify overall effect sizes across interventions. Future research should prioritise standardised outcome reporting, particularly for work-related outcomes, to improve comparability across studies.

## Conclusion

In conclusion, this review emphasises the need for holistic workplace interventions to manage CMSDs. Combining physical exercise with ergonomic, educational and digital strategies appears beneficial for improving clinical outcomes. However, the inconsistent definition and measurement of work-related outcomes across studies limits the ability to draw firm conclusions about their impact on occupational functioning. Future research should prioritise the use of standardised outcome definitions and measurement approaches, particularly for work-related outcomes, to improve comparability and strengthen the evidence base.

## Supplementary material

10.1136/bmjopen-2025-115276 online supplemental file 1

10.1136/bmjopen-2025-115276 online supplemental file 2

## Data Availability

All data relevant to the study are included in the article or uploaded as supplementary information.
