# Supplementary material for: Workplace interventions for chronic musculoskeletal disorders: a systematic review
Source: BMJ Open. 2026 Jun 22;16(6):e115276. doi: 10.1136/bmjopen-2025-115276 (PMC13288689; doi:10.1136/bmjopen-2025-115276)
Supplement: online supplemental file 1 [file bmjopen-16-6-s001.pdf]

Edit Search

Search Name

Comment

Type your comment here

Save Cancel

Set	Search Statement	Annotations	Insert	Edit	Delete
1.	employ*.ti,ab,kw.				
2.	worker*.ti,ab,kw.				
3.	exp Workforce/				
4.	"work force*".ti,ab,kw.				
5.	exp Workplace/				
6.	(staff* or personnel).ti,ab,kw.				
7.	"labo?r force".ti,ab,kw.				
8.	1 or 2 or 3 or 4 or 5 or 6 or 7				
9.	exp Musculoskeletal Diseases/				
10.	exp Musculoskeletal Pain/				
11.	exp Back Pain/				
12.	exp Neck Pain/				
13.	exp Low Back Pain/				
14.	exp Shoulder Pain/				
15.	exp Posture/				
16.	(MSK or musculoskeletal or "chronic musculoskeletal" or "musculoskeletal condition*" or "musculoskeletal disorder" or "MSK condition*" or MSD* or "chronic musculoskeletal condition" or condition* or disorder*).ti,ab,kw.				
17.	("knee pain" or "wrist pain" or "inflammatory arthritis" or "postural malalignment" or "Chronic illness" or "Arthritis - complications").ti,ab,kw.				
18.	9 or 10 or 11 or 12 or 13 or 14 or 15 or 16 or 17				
19.	(manage* or cope* or decrease* or improve* or control* or handle* or				

	avoid* or reduce* or stop* or "deal with" or (strateg* or tactic*) or intervention* or practice* or polic* or treatment* or approach* or method* or protocol* or process* or system*).ti,ab,kw.
20.	exp Ergonomics/
21.	exp Health Personnel/
22.	exp Exercise Therapy/
23.	exp Exercise/
24.	exp "Occupational Health"/
25.	exp Resistance Training/
26.	exp Health Promotion/
27.	exp Cognitive Behavioral Therapy/
28.	exp Pain Management/
29.	exp Motivational Interviewing/
30.	20 or 21 or 22 or 23 or 24 or 25 or 26 or 27 or 28 or 29
31.	(physiotherapy or "physical therapy" or flexibility or "flexible work" or "healthcare professional*" or ("exercise program" or "exercise programme*") or "health and safety" or "work conversation*" or "Physical activit*" or "workplace exercise*" or "occupational physical training" or " interval training" or "Physical training" or "Strengthening exercise*" or CBT or MI or "phase return").ti,ab,kw.
32.	30 or 31
33.	32 and 18 and 8
34.	from 33 keep 12,33,40,53,56,69,78,91,103,105,109,117,126-128,168,174,206,246,259,325,327,343,351,391,439,452,454,488,510,523,527,547,550,555,560,589-590,606,638,662,692,703,722,737,790,806,862,880,916,941-942,969,974,978,987,1002-1003,1005-1006,1014,1107,1194,1233,1284,1359,1421,1423,1429-1430,1438,1462-1463,1500,1504,1514,1529,1531-1532,1534,1540,1546,1551,1579,1599,1641,1689,1697,1705,1723,1756,1775,1778,1805,1812,1846,1873,1909,1916-1917,1921,1923,1939,1942,1970,2049,2052,2085,2095,2150,2153,2167,2172,2185,2200,2208-2209,2217,2232,2236,2263,2294,2301,2321,2380,2391,2405,2413,2418,2449-2450,2456,2487,2557,2582,2585,2633,2641,2655,2664,2713,2727,2776,2791,2800-2801,2806,2808,2830,2834,2849-2850,2857-2858,2905,2907,2920-2921,2948,2960,2970,2972,2999,3024,3030,3048,3055,3058,3065,3133,3210,3230,324

0,3264,3295,3321,3369,3383,3391,3418,343
2,3436-
3437,3444,3448,3462,3479,3489,3497,3500,
3515,3533-
3534,3545,3549,3562,3569,3607,3636,3643,
3703,3715,3744,3786,3804,3810,3851,3862,
3885,3963,3992,4062,4076,4093,4181,4204
,4251,4257,4259,4293,4298,4312,4337,4343,
4345,4352,4365,4382,4429,4437,4456,4476,
4497,4518,4602,4624,4656,4673,4690,4730
,4785,4811,4834,4845,4848,4893,4897,492
5,4929,4945,4969,4980,4994,4998,5006,5
035,5080,5085,5150,5166,5174,5181,5196,5
232,5294,5309,5347,5352,5368,5371,5381,5
383,5419,5425,5450,5453,5464,5514,5536-
5537,5541,5558,5575,5650-
5651,5659,5682,5688,5691,5756,5941,5955,
5983,5987,5995,6015,6035,6044,6056,6061
,6069,6081-
6082,6102,6132,6134,6151,6156,6161,6164,6
171,6211,6232,6259,6272,6289,6304,6365,6
390,6404,6439,6446,6455,6470,6474,6478,
6491,6604,6616,6648,6652,6733,6784-
6785,6794,6806,6808,6818,6821,6843,690
8,6940,6982,6985,6995-
6996,7002,7007,7015,7017,7041,7060,7065,
7067,7102,7116,7153,7162,7166,7169,7180,72
11,7249,7256,7295,7314,7317,7326,7354,7378
,7403,7415,7442,7457,7491,7541,7554-
7555,7617,7621,7624,7634,7655,7674,7679,76
99-7700,7709,7711-
7712,7722,7726,7767,7773,7775,7777,7791,779
9,7847-
7848,7850,7869,7881,7887,7889,7902,7905,
7907,7925,7947,7951,7957,7962,7980,8017,8
019,8024,8030-
8031,8076,8082,8087,8098,8112,8122,8151,
8164,8173,8210,8227,8244,8253,8266,8275,
8295,8305,8327,8359,8375,8377,8389,8400,
8424,8427,8452,8456,8470,8506,8509-
8510,8538,8553,8598,8630,8635,8646-
8647,8649,8664,8706-
8707,8719,8725,8755,8758,8769,8773,8775,8
788,8797,8800,8804,8815,8817,8819,8825,
8835,8855,8878,8915,8919,8927,8930,8933
,8957,8959,8992,8996,9050,9111,9123,9130
,9132,9134,9139,9141,9145,9153,9191,9222,
9230,9249,9254,9264,9272,9338,9400,9410
,9415,9418,9455,9494,9496,9505,9510,9517
,9550-
9551,9556,9584,9617,9642,9655,9702,9704,
9723-9724,9727,9730,9745-
9746,9758,9764,9766-9767,9831,9835-
9836,9856,9877,9888,9894,9910,9932,9942
,9949,9955-
9956,9962,9987,10017,10034,10037,10041,10
070,10076-10077,10093,10116-
10117,10153,10156,10169,10194,10206,10214,
10221,10239,10255,10259,10261,10271,10286,
10297,10316,10324,10328,10330,10366,10381
-10382,10405,10411,10414,10432-
10433,10459,10461,10489,10501,10514,10530

,10560,10569,10574,10576,10582,10585,1059
1,10611,10638,10640-
10642,10650,10656,10668,10672,10676,1068
0,10708,10713,10749,10777-
10778,10781,10809,10834,10845,10852,1088
9,10895,10934,10953,10963,10984,10991,110
04-11005,11009,11017,11024

#	Query	Limiters/Expanders	Last Run Via	Results
S29	S10 AND S14 AND S28	Limiters - Publication Year: 2018-2024; English Language; Publication Type: Randomized Controlled Trial<br>Expanders - Apply equivalent subjects<br>Search modes - Boolean/Phrase	Interface - EBSCOhost Research Databases<br>Search Screen - Advanced Search<br>Database - CINAHL Plus	83
S28	S15 OR S26	Expanders - Apply equivalent subjects<br>Search modes - Boolean/Phrase	Interface - EBSCOhost Research Databases<br>Search Screen - Advanced Search<br>Database - CINAHL Plus	319,760
S27	S10 AND S14 AND S15 AND S26	Expanders - Apply equivalent subjects<br>Search modes - Boolean/Phrase	Interface - EBSCOhost Research Databases<br>Search Screen - Advanced Search<br>Database - CINAHL Plus	0
S26	S16 OR S17 OR S18 OR S19 OR S20 OR S21 OR S22 OR S23 OR S24 OR S25	Limiters - Publication Year: 2018-2024<br>Expanders - Apply equivalent subjects<br>Search modes - Boolean/Phrase	Interface - EBSCOhost Research Databases<br>Search Screen - Advanced Search<br>Database - CINAHL Plus	319,745
S25	(MH "Motivational Interviewing")	Expanders - Apply equivalent subjects<br>Search modes - Boolean/Phrase	Interface - EBSCOhost Research Databases<br>Search Screen - Advanced Search<br>Database - CINAHL Plus	4,301
S24	(MH "Cognitive Therapy") OR (MH "Behavior Therapy")	Expanders - Apply equivalent subjects<br>Search modes - Boolean/Phrase	Interface - EBSCOhost Research Databases<br>Search Screen - Advanced Search<br>Database - CINAHL Plus	34,602

S23	(MH "Pain Management")	Expanders - Apply equivalent subjects<br>Search modes - Boolean/Phrase	Interface - EBSCOhost Research Databases<br>Search Screen - Advanced Search<br>Database - CINAHL Plus	16,622
S22	(MH "Resistance Training")	Expanders - Apply equivalent subjects<br>Search modes - Boolean/Phrase	Interface - EBSCOhost Research Databases<br>Search Screen - Advanced Search<br>Database - CINAHL Plus	7,505
S21	(MH "Health Promotion")	Expanders - Apply equivalent subjects<br>Search modes - Boolean/Phrase	Interface - EBSCOhost Research Databases<br>Search Screen - Advanced Search<br>Database - CINAHL Plus	81,169
S20	(MH "Occupational Therapy+")	Expanders - Apply equivalent subjects<br>Search modes - Boolean/Phrase	Interface - EBSCOhost Research Databases<br>Search Screen - Advanced Search<br>Database - CINAHL Plus	28,224
S19	(MH "Health Personnel+")	Expanders - Apply equivalent subjects<br>Search modes - Boolean/Phrase	Interface - EBSCOhost Research Databases<br>Search Screen - Advanced Search<br>Database - CINAHL Plus	659,531
S18	(MH "Ergonomics+")	Expanders - Apply equivalent subjects<br>Search modes - Boolean/Phrase	Interface - EBSCOhost Research Databases<br>Search Screen - Advanced Search<br>Database - CINAHL Plus	34,113
S17	(MH "Physical Therapy+")	Expanders - Apply equivalent subjects<br>Search modes - Boolean/Phrase	Interface - EBSCOhost Research Databases<br>Search Screen - Advanced Search<br>Database - CINAHL Plus	163,801
S16	(MH "Exercise+") OR (MH "Therapeutic Exercise+")	Expanders - Apply equivalent subjects<br>Search modes - Boolean/Phrase	Interface - EBSCOhost Research Databases<br>Search Screen - Advanced Search<br>Database - CINAHL Plus	158,746
S15	"manage* OR prevent* OR cope* OR decrease* OR improve* OR control*	Limiters - Publication Year: 2018-2024<br>Expanders - Apply	Interface - EBSCOhost Research Databases<br>Search Screen - Advanced	1,081

	OR handle* OR avoid* OR reduce* OR stop* OR deal with"	equivalent subjects<br>Search modes - SmartText Searching	Search<br>Database - CINAHL Plus	
S14	S11 OR S12 OR S13	Expanders - Apply equivalent subjects<br>Search modes - Boolean/Phrase	Interface - EBSCOhost Research Databases<br>Search Screen - Advanced Search<br>Database - CINAHL Plus	100,677
S13	"MSK OR musculoskeletal OR chronic musculoskeletal OR chronic MSK"	Limiters - Publication Year: 2018-2024<br>Expanders - Apply equivalent subjects<br>Search modes - SmartText Searching	Interface - EBSCOhost Research Databases<br>Search Screen - Advanced Search<br>Database - CINAHL Plus	511
S12	"msk conditions or msk disorders or musculoskeletal"	Limiters - Publication Year: 2018-2024<br>Expanders - Apply equivalent subjects<br>Search modes - SmartText Searching	Interface - EBSCOhost Research Databases<br>Search Screen - Advanced Search<br>Database - CINAHL Plus	510
S11	(MH "Musculoskeletal Diseases+") OR (MM "Low Back Pain") OR (MH "Musculoskeletal Pain") OR (MH "Back Pain") OR (MH "Knee Pain") OR (MH "Neck Pain")	Limiters - Publication Year: 2018-2024<br>Expanders - Apply equivalent subjects<br>Search modes - Boolean/Phrase	Interface - EBSCOhost Research Databases<br>Search Screen - Advanced Search<br>Database - CINAHL Plus	100,630
S10	S1 OR S2 OR S3 OR S4 OR S5 OR S6 OR S7 OR S8 OR S9	Limiters - Publication Year: 2018-2024; English Language<br>Expanders - Apply equivalent subjects<br>Search modes - Boolean/Phrase	Interface - EBSCOhost Research Databases<br>Search Screen - Advanced Search<br>Database - CINAHL Plus	174,334
S9	"work force"	Expanders - Apply equivalent subjects<br>Search modes - Boolean/Phrase	Interface - EBSCOhost Research Databases<br>Search Screen - Advanced Search<br>Database - CINAHL Plus	7,607
S8	"labour force OR labor force"	Expanders - Apply equivalent subjects	Interface - EBSCOhost Research Databases<br>Search Screen - Advanced	2,041

		Search modes - SmartText Searching	Search<br>Database - CINAHL Plus	
S7	"labour force OR labor force"	Expanders - Apply equivalent subjects<br>Search modes - Boolean/Phrase	Interface - EBSCOhost Research Databases<br>Search Screen - Advanced Search<br>Database - CINAHL Plus	0
S6	"personnel"	Expanders - Apply equivalent subjects<br>Search modes - Boolean/Phrase	Interface - EBSCOhost Research Databases<br>Search Screen - Advanced Search<br>Database - CINAHL Plus	275,224
S5	"staff"	Expanders - Apply equivalent subjects<br>Search modes - Boolean/Phrase	Interface - EBSCOhost Research Databases<br>Search Screen - Advanced Search<br>Database - CINAHL Plus	180,894
S4	(MH "Workforce")	Expanders - Apply equivalent subjects<br>Search modes - Boolean/Phrase	Interface - EBSCOhost Research Databases<br>Search Screen - Advanced Search<br>Database - CINAHL Plus	16,324
S3	"worker*"	Expanders - Apply equivalent subjects<br>Search modes - Boolean/Phrase	Interface - EBSCOhost Research Databases<br>Search Screen - Advanced Search<br>Database - CINAHL Plus	123,149
S2	"employee*"	Expanders - Apply equivalent subjects<br>Search modes - Boolean/Phrase	Interface - EBSCOhost Research Databases<br>Search Screen - Advanced Search<br>Database - CINAHL Plus	63,667
S1	"employer"	Expanders - Apply equivalent subjects<br>Search modes - Boolean/Phrase	Interface - EBSCOhost Research Databases<br>Search Screen - Advanced Search<br>Database - CINAHL Plus	16,964

Scopus

Edit

(TITLE-ABS-KEY (employer OR employee* OR worker* OR (workforce OR work AND force) OR staff OR personnel OR ("labour force" OR "labor force"))) AND (TITLE-ABS-KEY ((msk OR musculoskeletal OR "chronic msk" OR "chronic musculoskeletal" OR "musculoskeletal condition*" OR "musculoskeletal disorder*" OR "msk condition*" OR msd* OR "chronic musculoskeletal condition*" OR "chronic musculoskeletal disorder*" OR condition* OR disorder* OR "chronic pain" OR "musculoskeletal pain" OR "back pain" OR "neck pain" OR "low back pain" OR " shoulder pain" OR "knee pain" OR "wrist pain" OR arthritis OR "inflammatory arthritis" OR "postural malalignment" OR "posture" OR "chronic illness" OR "arthritis - complications" OR "chronic musculoskeletal disorders "))) AND (TITLE-ABS-KEY ((manage AND * OR cope* OR decrease* OR improve* OR control* OR handle* OR avoid* OR reduce* OR stop* OR "deal with" OR (strateg* OR tactic*) OR intervention* OR practice* OR polic* OR treatment* OR plan* OR approach* OR method* OR protocol* OR process* OR system*))) AND (TITLE-ABS-KEY ((exercise* OR physiotherapy OR "physical therapy" OR ergonomic* OR flexibility OR "flexible work" OR "healthcare professional*" OR "health personnel" OR " exercise program" OR "exercise programme*" OR "exercise therapy" OR "occupational health" OR "health safety" OR "work conversation*" OR "physical activit*" OR "exercise therap*" OR "workplace exercise*" OR "occupational physical training" OR "interval training" OR "physical training" OR "resistance training" OR "strengthening exercise" OR "strengthening exercise*" OR "health promotion" OR cbt OR "pain management" OR "cognitive behavioural therapy" OR mi OR "motivational interviewing" OR "phase return"))) AND (LIMIT-TO (DOCTYPE , "ar")) AND (LIMIT-TO (LANGUAGE , "english")) AND (LIMIT-TO (SRCTYPE , "j")) AND (EXCLUDE (EXACTKEYWORD , "attitude of health personnel") OR EXCLUDE (EXACTKEYWORD , "health personnel attitude") OR EXCLUDE (EXACTKEYWORD , "qualitative research") OR EXCLUDE (EXACTKEYWORD , "cross-sectional study") OR EXCLUDE (EXACTKEYWORD , "surveys and questionnaires") OR EXCLUDE (EXACTKEYWORD , "attitude to health") OR EXCLUDE (EXACTKEYWORD , "health knowledge, attitudes, practice") OR EXCLUDE (EXACTKEYWORD , "retrospective studies") OR EXCLUDE (EXACTKEYWORD , "systematic review")) AND (LIMIT-TO (PUBSTAGE , "final")

Search History (35)

View Saved

# ▲	Searches	Results	Type	Actions		Annotations
1	(employ* or worker* or "work force*" or staff*).mp. [mp=title, abstract, heading word, table of contents, key concepts, original title, tests & measures, mesh word]	496854	Advanced	Display Results	More	
2	(workplace or "work force" or "labo?r force").mp. [mp=title, abstract, heading word, table of contents, key concepts, original title, tests & measures, mesh word]	58750	Advanced	Display Results	More	
3	*Personnel/	6605	Advanced	Display Results	More	
4	1 or 2 or 3	518994	Advanced	Display Results	More	
5	exp Musculoskeletal Disorders/	21565	Advanced	Display Results	More	
6	pain/ or *back pain/ or *chronic pain/	48759	Advanced	Display Results	More	
7	("musculoskeletal disease*" or "musculoskeletal pain" or "musculoskeletal condition" or "musculoskeletal disorder" or MSK or "chronic musculoskeletal" or "chronic musculoskeletal condition").mp. [mp=title, abstract, heading word, table of contents, key concepts, original title, tests & measures, mesh word]	3929	Advanced	Display Results	More	
8	*Posture/	5695	Advanced	Display Results	More	
9	("neck pain" or "shoulder pain" or "knee pain" or "low* back pain").mp. [mp=title, abstract, heading word, table of contents, key concepts, original title, tests & measures, mesh word]	7274	Advanced	Display Results	More	
10	5 or 6 or 7 or 8 or 9	73983	Advanced	Display Results	More	
11	*Arthritis/	2447	Advanced	Display Results	More	
12	(employ* or worker* or "work force*" or staff*).mp. [mp=title, abstract, heading word, table of contents, key concepts, original title, tests & measures, mesh word]	496854	Advanced	Display Results	More	
13	(workplace or "work force" or "labo?r force").mp. [mp=title, abstract, heading word, table of contents, key concepts, original title, tests & measures, mesh word]	58750	Advanced	Display Results	More	
14	*Personnel/	6605	Advanced	Display Results	More	
15	12 or 13 or 14	518994	Advanced	Display Results	More	
16	exp Musculoskeletal Disorders/	21565	Advanced	Display Results	More	
17	pain/ or *back pain/ or *chronic pain/	48759	Advanced	Display Results	More	
18	("musculoskeletal disease*" or "musculoskeletal pain" or "musculoskeletal condition" or "musculoskeletal disorder" or MSK or "chronic musculoskeletal" or "chronic musculoskeletal condition").mp. [mp=title, abstract, heading word, table of contents, key concepts, original title, tests & measures, mesh word]	3929	Advanced	Display Results	More	
19	*Posture/	5695	Advanced	Display Results	More	

20	("neck pain" or "shoulder pain" or "knee pain" or "low* back pain").mp. [mp=title, abstract, heading word, table of contents, key concepts, original title, tests & measures, mesh word]	7274	Advanced	Display Results	More	
21	*Arthritis/	2447	Advanced	Display Results	More	
22	16 or 17 or 18 or 19 or 20 or 21	73983	Advanced	Display Results	More	
23	(manage* or cope* or decrease* or improve* or control* or handle* or avoid* or reduce* or stop* or "deal with" or (strateg* or tactic*) or intervention* or practice* or polic* or treatment* or approach* or method* or protocol* or process* or system*).mp. [mp=title, abstract, heading word, table of contents, key concepts, original title, tests & measures, mesh word]	4020974	Advanced	Display Results	More	
24	exp Health Personnel/	199490	Advanced	Display Results	More	
25	*Exercise Therapy/	665	Advanced	Display Results	More	
26	exp Exercise/	33544	Advanced	Display Results	More	
27	exp Occupational Health/	5878	Advanced	Display Results	More	
28	exp Cognitive Behavior Therapy/	56489	Advanced	Display Results	More	
29	exp Pain Management/	11856	Advanced	Display Results	More	
30	exp Motivational Interviewing/	3204	Advanced	Display Results	More	
31	exp Physical Therapy/	4379	Advanced	Display Results	More	
32	("ergonomic*" or "resistance training*" or "health promotion" or physiotherapy or "exercise program*" or "exercise programme*" or "work conversation*" or "occupational physical training" or "interval training" or "strengthening exercise*" or CBT or MI or "phase return").mp. [mp=title, abstract, heading word, table of contents, key concepts, original title, tests & measures, mesh word]	84463	Advanced	Display Results	More	
33	24 or 25 or 26 or 27 or 28 or 29 or 30 or 31 or 32	361621	Advanced	Display Results	More	
34	15 and 22 and 23 and 33	2064	Advanced	Display Results	More	
35	limit 34 to yr="2018 - 2024"	607	Advanced	Display Results	More	

Save Remove Combine with: AND OR Contract

Save All Create RSS Create Auto-Alert View Saved Share Search History

Basic Search Find Citation Search Tools Search Fields Advanced Search Multi-Field Search

1 resource selected Hide Change

APA PsycInfo 1967 to May Week 4 2024

Keyword Author Title Journal

Enter keyword or phrase (* or $ for truncation)

Search

Term Finder

Include Multimedia Map Term to Subject Heading

Limits

- Full Text
- Latest Update
- Abstracts
- Impact Statement
- APA PsycArticles Journals
- Human
- Test DOI
- Remove MEDLINE Records
- All Journals
- English Language
- Open Access

Publication Year - -

Additional Limits Edit Limits

Hide Options Print Email Export + My Projects Keep Selected

Search Information

You searched:
limit 34 to yr="2018 - 2024"

Search terms used:
approach*
arthritis
avoid*
back
pain
cbt
chronic
musculoskeletal
condition
cognitive
behavior
therapy
control*
cope*
deal
with
decrease*
employ*
ergonomic*
exercise
program*
programme*
handle*
health
personnel
promotion
improve*
interval
training
intervention*
knee
labo?r
force
low*
manage*
method*
mi
motivational
interviewing
msk
disease*
disorder
disorders
neck
occupational
physical
management
phase
return
physiotherapy
polic*
posture
practice*
process*
protocol*

All Range Ex: 1-4, 7 Clear View: Results per page 10 Go to result 1 Go

Next

1. **The impact of multiple regions of pain and work-life balance among healthcare workers. [References].**

Kesiena, Onoriode; Atarere, Joseph; Benden, Mark.

Work: Journal of Prevention, Assessment & Rehabilitation. Vol.75,(1), 2023, pp. 357-362.

[Journal; Peer Reviewed Journal]

Year of Publication
2023

Abstract Cite + My Projects + Annotate

Abstract Reference Complete Reference Find Similar Find Citing Articles FindIt@Bham

2. **The effects of biomechanical risk factors on musculoskeletal disorders among baggers in the supermarket industry. [References].**

Gumasing, Ma. Janice J; Prasetyo, Yogi Tri; Jaurigue, Jenile; Saavedra, Daphne Nicole M; Nadlifatin, Reny; Chuenyindee, Thanatorn; Persada, Satria Fadil.

Work: Journal of Prevention, Assessment & Rehabilitation. Vol.75,(1), 2023, pp. 315-324.

[Journal; Peer Reviewed Journal]

Year of Publication
2023

Abstract Cite + My Projects + Annotate

Abstract Reference Complete Reference Find Similar Find Citing Articles FindIt@Bham

3. **Hand-arm vibration syndrome among harvesting farmers: A cross-sectional study from Pakistan. [References].**

Kashif, Muhammad; Talib, Azka; Imtiaz, Zainab; Imtiaz, Zara; Dustgir, Atif; Syed, Hafiza Aroosa.

Work: Journal of Prevention, Assessment & Rehabilitation. Vol.75,(1), 2023, pp. 265-273.

[Journal; Peer Reviewed Journal]

Year of Publication
2023

Abstract Reference Complete Reference Find Similar Find Citing Articles FindIt@Bham

reduce*
resistance
training*
shoulder
staff*
stop*
strateg*
strengthening
exercise*
system*
tactic*
treatment*
work
conversation*
force*
worker*
workplace

Search Returned:
607 text results

Sort By:

-

Customize Display

Filter By

Add to Search History

Selected Only (0)

Years

All Years

Current year

Past 3 years

Past 5 years

Specific Year Range

From: ex: 2009 To: ex: 2009 Apply

Subject

All Subjects

Pain

Musculoskeletal Disorders

Human Factors Engineering

Back Pain

Occupational Health

More ...

Author

Journal

All Journals

Work: Journal of Prevention, Assessment & Rehabilitation

Dissertation Abstracts International: Section B: The Sciences and Engineering

Journal of Occupational Rehabilitation

Applied Ergonomics

Human Factors

More ...

Book

Publication Type

Abstract Cite + My Projects

+ Annotate

---

4. **What affects musculoskeletal risk in nursing assistants and orderlies? [References].**

Oliver-Hernandez, Coral; Li, Shimeng; Astudillo, Raul Jimenez; Rodriguez, Inmaculada Mateo.

Work: Journal of Prevention, Assessment & Rehabilitation. Vol.75,(1), 2023, pp. 145-155.

[Journal; Peer Reviewed Journal]

Year of Publication
2023

Abstract Cite + My Projects

+ Annotate

Abstract Reference
Complete Reference
Find Similar
Find Citing Articles
FindIt@Bham

---

5. **Acute effects of a warm-up intervention on pain, productivity, physical capacities and psychological perceptions among vineyard workers: A cluster randomized trial. [References].**

Larinier, Nicolas; Vuillerme, Nicolas; Jadaud, Alexandre; Malherbe, Solene; Giraud, Eymeric; Balaguier, Romain.

Journal of Occupational Rehabilitation. Vol.34,(1), 2024, pp. 100-115.

[Journal; Peer Reviewed Journal]

Year of Publication
2024

Publication Month/Season
Mar

Abstract Cite + My Projects

+ Annotate

Abstract Reference
Complete Reference
Find Similar
Find Citing Articles
FindIt@Bham

---

6. **Considerations on the obstacles that lead to slow recruitment in a pain management clinical trial: Experiences from the Belgian PELICAN (PrEgabalin Lidocaine Capsaicin Neuropathic Pain) pragmatic study. [References].**

Hans, Guy H; Almeshal, Dima; Vanlommel, Lotte; Roelant, Ella; Verhaegen, Iris; Smits, Elke; Van Boxem, Koen; Fontaine, Robert.

Pain Research & Management. Vol.2023, 2023, ArtID 7708982.

[Journal; Peer Reviewed Journal]

Year of Publication
2023

Publication Month/Season
Apr

Abstract Reference
Complete Reference
Find Similar
Find Citing Articles
Full Text

All Types

Journal

Peer Reviewed Journal

Dissertation Abstract

Book

Edited Book

My Projects

+ New Project

No projects available.

Abstract Cite + My Projects

+ Annotate

7. **Environmental risk assessment of low back pain in ICU nurses: An instrument development study. [References].**

Zhang, Lihui; Liu, Yangyang; Yuan, Su'e.

Journal of Nursing Management. Vol.2023, 2023, ArtID 3649293.

[Journal; Peer Reviewed Journal]

Year of Publication

2023

Publication Month/Season

Apr

Abstract Cite + My Projects

+ Annotate

Abstract Reference

Complete Reference

Find Similar

Find Citing Articles

FindIt@Bham

8. **Durability of the treatment effects of an 8-week self-administered home-based virtual reality program for chronic low back pain: Follow-up study of a randomized clinical trial. [References].**

Garcia, Laura; Birckhead, Brandon; Krishnamurthy, Parthasarathy; Mackey, Ian; Sackman, Josh; Salmasi, Vafi; Louis, Robert; Castro, Carina; Maddox, Roselani; Maddox, Todd; Darnall, Beth D.

Journal of Medical Internet Research. Vol.24,(5), 2022, pp. 703-720.

[Journal; Peer Reviewed Journal]

Year of Publication

2022

Publication Month/Season

May

Abstract Cite + My Projects

+ Annotate

Abstract Reference

Complete Reference

Find Similar

Find Citing Articles

FindIt@Bham

9. **Findings and guidelines on provider technology, fatigue, and well-being: Scoping review. [References].**

Hilty, Donald M; Armstrong, Christina M; Smout, Shelby A; Crawford, Allison; Maheu, Marlene M; Drude, Kenneth P; Chan, Steven; Yellowlees, Peter M; Krupinski, Elizabeth A.

Journal of Medical Internet Research. Vol.24,(5), 2022, pp. 1-15.

[Journal; Peer Reviewed Journal]

Year of Publication

2022

Publication Month/Season

May

Abstract Reference

Complete Reference

Find Similar

Find Citing Articles

FindIt@Bham

Abstract Cite + My Projects

+ Annotate

10. **Changes in resting-state brain activity after cognitive behavioral therapy for chronic pain: A magnetoencephalography study.**

Yoshino, Atsuo; Maekawa, Toru; Kato, Miyuki; Chan, Hui-Ling; Otsuru, Naofumi; Yamawaki, Shigeto.

The Journal of Pain. 2024, pp. No Pagination Specified.

[Journal; Peer Reviewed Journal]

Year of Publication
2024

Publication Month/Season
Apr

Abstract Cite + My Projects

+ Annotate

Abstract Reference

Complete Reference

Find Similar

Find Citing Articles

FindIt@Bham

Range
All Ex: 1-4, 7 Clear View:
Results per page 10
Go to result 1 Go

Print Email Export + My Projects Keep Selected

Search History/Alerts

Print Search History Retrieve Searches Retrieve Alerts Save Searches / Alerts

Select / deselect all | Search with AND | Search with OR | Delete Searches | Refresh Search Results

Search ID#	Search Terms	Search Options	Actions
S14	S8 AND S9 AND S11	**Limiters** - Publication Date: 20180101-20241231<br><br>**Expanders** - Apply equivalent subjects<br><br>**Narrow by Language:** - english<br><br>**Search modes** - Boolean/Phrase	Rerun<br>View Details<br>Edit
S13	S8 AND S9 AND S11	**Limiters** - Publication Date: 20180101-20241231<br><br>**Expanders** - Apply equivalent subjects<br><br>**Search modes** - Boolean/Phrase	Rerun<br>View Details<br>Edit

☐	S12	S8 AND S9 AND S11	**Expanders** - Apply equivalent subjects<br><br>**Search modes** - Boolean/Phrase	Rerun<br>View Details<br>Edit
☐	S11	(exercise* OR physiotherapy OR "physical therapy" OR ergonomic* OR flexibility OR "flexible work" OR "healthcare professional*" OR "health personnel" OR " exercise program" OR "exercise programme*" OR "exercise therapy" OR "occupational health" OR "health safety" OR "work conversation*" OR "physical activit*" OR "exercise therap*" OR "workplace exercise*" OR "occupational physical training" OR "interval training" OR "physical training" OR "resistance training" OR "strengthening exercise" OR "st ...	**Expanders** - Apply equivalent subjects<br><br>**Search modes** - Boolean/Phrase	Rerun<br>View Details<br>Edit
☐	S10	(manage AND * OR cope* OR decrease* OR improve* OR control* OR handle* OR avoid* OR reduce* OR stop* OR "deal with" OR (strateg* OR tactic*) OR intervention* OR practice* OR polic* OR treatment* OR plan* OR approach* OR method* OR protocol* OR process* OR system*)	**Expanders** - Apply equivalent subjects<br><br>**Search modes** - SmartText Searching	Rerun<br>View Details<br>Edit
☐	S9	(msk OR musculoskeletal OR "chronic msk" OR "chronic musculoskeletal" OR "musculoskeletal condition*" OR "musculoskeletal disorder*" OR "msk condition*" OR msd* OR "chronic musculoskeletal condition*" OR "chronic musculoskeletal disorder*" OR condition* OR disorder* OR "chronic pain" OR "musculoskeletal pain" OR "back pain" OR "neck pain" OR "low back pain" OR " shoulder pain" OR "knee pain" OR "wrist pain" OR arthritis OR "inflammatory arthritis" OR "postural malalignment" OR "posture" OR "chr ...	**Expanders** - Apply equivalent subjects<br><br>**Search modes** - Boolean/Phrase	Rerun<br>View Details<br>Edit
☐	S8	(employer OR employee* OR worker* OR (workforce OR work AND force) OR staff OR personnel OR ("labour force" OR "labor force")	**Expanders** - Apply equivalent subjects<br><br>**Search modes** - Boolean/Phrase	Rerun<br>View Details<br>Edit
☐	S7	S1 AND S2 AND S4	**Limiters** - Publication Date:	View Results (284)

			20180101-20241231<br><br>**Expanders** - Apply equivalent subjects<br><br>**Narrow by Language:** - english<br><br>**Search modes** - Boolean/Phrase	View Details<br>Edit
☐	S6	S1 AND S2 AND S4	**Limiters** - Publication Date: 20180101-20241231<br><br>**Expanders** - Apply equivalent subjects<br><br>**Search modes** - Boolean/Phrase	View Results (285)<br>View Details<br>Edit
☐	S5	S1 AND S2 AND S4	**Expanders** - Apply equivalent subjects<br><br>**Search modes** - Boolean/Phrase	View Results (1,967)<br>View Details<br>Edit
☐	S4	(exercise* OR physiotherapy OR "physical therapy" OR ergonomic* OR flexibility OR "flexible work" OR "healthcare professional*" OR "health personnel" OR " exercise program" OR "exercise programme*" OR "exercise therapy" OR "occupational health" OR "health safety" OR "work conversation*" OR "physical activit*" OR "exercise therap*" OR "workplace exercise*" OR "occupational physical training" OR "interval training" OR "physical training" OR "resistance training" OR "strengthening exercise" OR "st ...	**Expanders** - Apply equivalent subjects<br><br>**Search modes** - Boolean/Phrase	View Results (74,051)<br>View Details<br>Edit
☐	S3	(manage AND * OR cope* OR decrease* OR improve* OR control* OR handle* OR avoid* OR reduce* OR stop* OR "deal with" OR (strateg* OR	**Expanders** - Apply equivalent	View Results (2)

		tactic*) OR intervention* OR practice* OR polic* OR treatment* OR plan* OR approach* OR method* OR protocol* OR process* OR system*)	subjects<br><br>**Search modes** - SmartText Searching	View Details<br>Edit
☐	S2	(msk OR musculoskeletal OR "chronic msk" OR "chronic musculoskeletal" OR "musculoskeletal condition*" OR "musculoskeletal disorder*" OR "msk condition*" OR msd* OR "chronic musculoskeletal condition*" OR "chronic musculoskeletal disorder*" OR condition* OR disorder* OR "chronic pain" OR "musculoskeletal pain" OR "back pain" OR "neck pain" OR "low back pain" OR " shoulder pain" OR "knee pain" OR "wrist pain" OR arthritis OR "inflammatory arthritis" OR "postural malalignment" OR "posture" OR "chr ...	**Expanders** - Apply equivalent subjects<br><br>**Search modes** - Boolean/Phrase	View Results (92,598)<br>View Details<br>Edit
☐	S1	(employer OR employee* OR worker* OR (workforce OR work AND force) OR staff OR personnel OR ("labour force" OR "labor force")	**Expanders** - Apply equivalent subjects<br><br>**Search modes** - Boolean/Phrase	View Results (16,875)<br>View Details<br>Edit
