# Supplementary material for: Workplace interventions for chronic musculoskeletal disorders: a systematic review
Source: BMJ Open. 2026 Jun 22;16(6):e115276. doi: 10.1136/bmjopen-2025-115276 (PMC13288689; doi:10.1136/bmjopen-2025-115276)
Supplement: online supplemental file 2 [file bmjopen-16-6-s002.docx]

**Table 4:** Full Study Characteristics

| **EXERCISE-BASED INTERVENTIONS** | | | | | | | | | |
| --- | --- | --- | --- | --- | --- | --- | --- | --- | --- |
| **Author** | **Country** | **Study design** | **Sample size (baseline & follow-up)** | **Age in years,**  **(mean)** | **Diagnosis** | **Occupation** | **Included Interventions** | **Outcomes (measurement instruments)** | **Intervention provider, frequency, duration, Follow-up** |
| Alqhtani et al [23] | Saudi Arabia | RCT | Baseline:  IG=30,  CG=30  Follow-up:  IG=30, CG=30 | 37.15 ± 3.9 | Various chronic MSDS | Healthcare professionals | I_g_: Whole-body stretching (WBS) exercises  C_g_: Education programme | Pain, functional disability (VAS, ODI) | **Provider**  Physiotherapists  **Frequency**  3 times/week  **Duration**  30 minutes  **Follow-up**  6 weeks |
| Alvani et al [19] | Iran | RCT | Baseline: IG=15, CG=15  Follow-up: IG=15, CG=15 | 35.64 ±6.99  I_g_=40.6± 6.03  C_g_= 30.67± 7.84 | Chronic low back pain | Military personnel | I_g_: Neuromuscular exercises including warm-up, specific exercises for stability, balance, and strength, and cool-down  C_g_: Routine physical activities without additional interventions | Pain, functional disability (VAS, ODI) | **Provider**  Exercise physiologists  **Frequency**  3 times/week  **Duration**  60 minutes  **Follow-up**  8 weeks |
| Beltrán et al [31] | Brazil | RCT | Baseline: IG=22, CG=22  Follow-up: N=42 | 38.97±9.18  I_g_= 37.68± 9.93  C_g_= 40.27± 8.39 | Chronic shoulder pain | Fruit workers | I_g_: Supervised workplace exercise programme  C_g_: Unsupervised stretching exercises using booklets | Pain, shoulder function (NPRS, SPADI) | **Provider**  Physiotherapist and a physical education teacher  **Frequency**  2 times/week  **Duration**  20-28 minutes  **Follow-up**  8 weeks |
| Bernardelli et al [12] | Italy | RCT | Baseline: IG=47, CG=37  Follow-up: N=84 | 51.12 ± 9.03  I_g_= 50.5 ± 9.7  C_g_= 51.9 ± 8.1 | Chronic low back pain | Health care workers | I_g_: Supervised workplace exercises  C_g_: Unsupervised home exercises using booklets and videos | Pain, functional disability (VAS, RMDQ) | **Provider**  Physiotherapist  **Frequency**  1 time/week  **Duration**  30 minutes  **Follow-up**  7 weeks |
| Ghavamabadi et al [18] | Iran | RCT | Baseline: IG=125, CG=125  Follow-up: IG=125, CG=125 | The study provides age ranges. | Chronic low back pain | Industrial workers | I_g_: Back School programme and education  C_g_: No training | Pain, functional disability (VAS, ODI) | **Provider**  Ergonomist  **Frequency**  1 time/week  **Duration**  2 hours  **Follow-up**  8 weeks |
| Jakobsen et al [17] | Denmark | C-RCT | Baseline: IG=111, CG=89  Follow-up: N=184 | 41.78 ±11.15  I_g_= 40 ±12  C_g_= 44 ±10 | Various chronic MSDS | Health care workers | I_g_: Group-based, supervised strength training during working hours  C_g_: Physical exercise at home | Pain, work ability (VAS, Work Ability Index) | **Provider**  Training instructor  **Frequency**  5 times/week  **Duration**  30-45 minutes  **Follow-up**  10 Weeks |
| Kang et al [24] | South Korea | RCT | Baseline: IG=15, CG=15  Follow-up: N=29 | 47.28 ± 4.32  I_g_=46.7 ± 4.6  C_g_=47.9±4.0 | Hand osteoarthritis | Automobile manufacturing workers | I_g_: Finger exercise programme combined with paraffin baths  C_g_: Paraffin baths | Pain, hand function (VAS, AUSCAN) | **Provider**  Physiotherapist  **Frequency**  5 times/week  **Duration**  30 minutes  **Follow-up**  8 Weeks |
| Karatrantou et al [25] | Greece | RCT | Baseline: IG=35, CG=35  Follow-up: N=70 | 44±6.37  I_g_=43.9 ± 6.8  C_g_=44.1 ± 5.9 | Various chronic MSDS | Office workers | I_g_: A workplace exercise programme focusing on flexibility, strength, and balance  C_g_: No intervention | Pain, functional disability (VAS, ODI) | **Provider**  Exercise specialists  **Frequency**  5 **times/week**  **Duration**  25-38 **minutes**  **Follow-up**  6 months |
| Korshøj et al [32] | Denmark | C-RCT | Baseline: IG=57, CG=59  Follow-up: IG=57, CG=59 | 45.3 ± 8.6  I_g_= 44.9 ± 9.2  C_g_=45.7 ±8.1 | Various CMSDs | Worksite cleaners | I_g_: Aerobic exercises  C_g_: Lectures on health promotion | Pain, work ability (VAS, Work Ability Index) | **Provider**  Exercise trainers  **Frequency**  2 times/week  **Duration**  30 minutes  **Follow-up**  4, 12 months |
| Moreira et al [27] | Brazil | RCT | Baseline: IG=46, CG=44  Follow-up: IG=46, CG=44 | 34.82±8.83  I_g_=37.53±9.93  C_g_=32.11±7.57 | Various CMSDs | Nursing assistants | I_g_: Supervised therapeutic exercise programme  C_g_: No intervention | Pain, quality of life (VAS, SF-36) | **Provider**  Physiotherapists  **Frequency**  2 times/week  **Duration**  30 minutes  **Follow-up**  12 Weeks |
| Passos et al [31] | Brazil | RCT | Baseline: IG=22, CG=22  Follow-up: N=44 | 37±9  I_g_= 38±9  C_g_=36±10 | Chronic low back pain | Fruit workers | I_g_: Supervised strength and flexibility exercises  C_g_: Booklet with guidelines for home exercises | Pain, functional disability (NPRS, ODI) | **Provider**  Physiotherapists  **Frequency**  2 times/week  **Duration**  20-28 minutes  **Follow-up**  8 Weeks |
| Phattharasupharerk et al [33] | Thailand | RCT | Baseline: IG=36, CG=36  Follow-up: N=65 | 35.25 ±3.97  I_g_= 35.7±3.6  C_g_= 34.8 ±4.30 | Chronic low back pain | Office workers | I_g_: Qigong exercises sessions  C_g_: General advice on  managing low back pain | Pain, functional disability (VAS, ODI) | **Provider**  Qigong instructors  **Frequency**  1 times/day  **Duration**  43 minutes  **Follow-up**  6 Weeks |
| Makki et al [22] | Iran | RCT | Baseline: IG=33, CG=33  Follow-up: N=66 | 42.8 ± 6.3  I_g_=40.21±8.59  C_g_= 38.33±7.7 | Various work related CMSDs | Office Workers | I_g_: Corrective exercise training (online)  C_g_: No intervention | Pain, posture, functional outcomes (VAS, posture analysis tools) | **Provider**  Online/Software  **Frequency**  Multiple times/day  **Duration**  Unclear  **Follow-up**  4 Weeks |
| **ERGONOMIC INTERVENTIONS** | | | | | | | | | |
| **Author** | **Country** | **Study design** | **Sample size (baseline& follow-up)** | **Age in years,**  **(mean)** | **Diagnosis** | **Occupation** | **Included Interventions** | **Outcomes (measurement instruments)** | **Intervention provider, frequency, duration, Follow-up** |
| Lee et al [15] | Brazil | RCT | Baseline: IG=32, CG=32  Follow-up: N=64 | 28.95 ± 6.36  I_g_=29.1 ± 7.9  C_g_=28.8± 4.3 | Various chronic MSDS | Office workers | I_g_: Ergonomic workstation adjustments  C_g_: Standard workplace setup | Pain, musculoskeletal symptoms (VAS, Nordic Musculoskeletal Questionnaire) | **Provider**  Unclear  **Frequency**  N/A  **Duration**  N/A  **Follow-up**  12, 24, and 36 Weeks |
| Yaghoubitajani et al [26] | Iran | RCT | Baseline: IG1=12, IG2=12, CG=12  Follow-up: IG1=12, IG2=12, CG=12 | 38.16±6.7  I_g1_= 38.91 ± 3.87  I_g2_= 38.58 ± 7.34  C_g_= 37.00 ± 8.12 | Upper Crossed Syndrome | Office workers | I_g1_: Corrective exercises (online)  I_g2_: Corrective exercises (at work)  C_g_: No intervention | Posture, musculoskeletal alignment (postural assessment tools) | **Provider**  Exercise expert  **Frequency**  3times per day  **Duration**  50–60 minutes  **Follow-up**  8 weeks |
| **MULTICOMPONENT INTERVENTIONS** | | | | | | | | | |
| **Author** | **Country** | **Study design** | **Sample size (baseline& follow-up)** | **Age in years,**  **(mean)** | **Diagnosis** | **Occupation** | **Included Interventions** | **Outcomes (measurement instruments)** | **Intervention provider, frequency, duration, length of observation** |
| Alshehre et al [14] | Saudi Arabia | RCT | Baseline: IG=12, CG=12  Follow-up: N=24 | 43.15±4.65  I_g_=42.1±4.5  C_g_=44.2±4.8 | Chronic neck pain | Office Workers | I_g_: Basic Body Awareness Therapy (BBAT), Neck-specific training exercises, and ergonomic modifications  C_g_: Ergonomic modifications | Pain, disability, range of motion (VAS, Neck Disability Index) | **Provider**  Physiotherapists  **Frequency**  2 times/week  **Duration**  60 minutes for BBAT  **Follow-up**  8 weeks |
| Hergenroeder et al [34] | USA | RCT | Baseline: IG=12, CG=12  Follow-up: N=24 | 50.15 ± 10.98  I_g_=51.5 ± 9.2  C_g_=48.8 ± 12.5 | Chronic low back pain | Desk workers | I_g_: Behavioural counseling, a sit-stand desk attachment, and an activity prompter aimed at reducing sedentary behaviour  C_g_: No intervention | Pain, physical activity, sedentary behaviour (VAS, activity monitoring tools) | **Provider**  Physiotherapist  **Frequency**  monthly  **Duration**  1.5-hour initial session,  14-minute calls  **Follow-up**  6 months |
| Imai et al [16] | Japan | RCT | Baseline: IG=51, CG=53  Follow-up: IG=51, CG=53 | The study provides age ranges. | Various chronic MSDS | Health care workers | I_g_: Pain Neuroscience Education (PNE) and exercise regimen tailored to individual healthcare workers  C_g_: Feedback-based programme without exercise | Pain, work productivity, quality of life (VAS, Work Productivity and Activity Impairment Questionnaire) | **Provider**  Physiotherapist  **Frequency**  3-4 times/week  **Duration**  50-60 minutes  **Length**  6 months |
| Rantonen et al [28] | Finland | RCT | Baseline: IG1=43, IG2=43, IG3=40, CG=50  Follow-up: same as baseline | 46.5 ± 8.7  I_g1_=45±9  I_g2_=44±8  I_g3_=45±7  C_g_=46±7 | Moderate low back pain | Forestry company | I_g1_: Multidisciplinary rehabilitation at the hospital  I_g2_: Progressive back exercises in outpatient Physiotherapy clinic  I_g3_: Self-care advice by workplace occupational physician  C_g_: No intervention | Pain, work ability (VAS, disability not clearly specified) | **Provider**  Various healthcare professionals depending on the group  **Frequency**  I_g1_: 2-3 times/week  I_g2_: 2-3 times/week  I_g3_: 1 session  **Duration**  I_g1_,_2_:60 minutes  I_g3_:80 minutes  **Follow-up**  24 months |
| Shariat et al [13] | Malaysia | RCT | Baseline: IG1=43, IG2=37, IG3=34, CG=28  Follow-up: N=142 | 29.5 ± 1.09  I_g1_=29.41±1.16  I_g2_=28.31±  0.92  I_g3_=29.64±0.90  C_g_=28.74 ±0.82 | Various MSDS | Office workers | I_g1_: Exercise training  I_g2_: Ergonomic modification  I_g3_: Combined exercise & modification  C_g_: No intervention | Pain, posture, musculoskeletal symptoms (VAS, posture assessment tools) | **Provider**  Exercise specialist, Occupational Health expert  **Frequency**  I_g1_: 3 times/week  I_g2_: 1 session  I_g3_: 2-3 times/week and 1 ergonomic session  **Duration**  60 minutes  **Follow-up**  6 months |
| Ting et al [29] | Australia | C-  RCT | Baseline: IG1=52, IG2=45  Follow-up: Not reported | 41.42±9.63  I_g1_= 41.37±8.41  I_g2_ = 41.46±10.88 | Chronic neck pain | Office Workers | I_g1_: Ergonomics, neck and shoulder strengthening exercises, and health promotion  I_g2_: Ergonomics and health promotion | Pain, functional disability (VAS, Neck Disability Index) | **Provider:** Physiotherapist  **Frequency:** 3 times/week  **Duration:**  20 min/session  **Data collection time:** 12 weeks and 12 months |
| Trøstrup et al [30] | Denmark | C-  RCT | Baseline: IG=57, CG=52  Follow-up: N=93 | 47.3±10.1  I_g_= 48.8±  9.5  C_g_= 45.7±  10.8 | Chronic shoulder pain | Manufacture, Construction | I_g_: Three group- café meetings with discussions, education, supervised exercises, workplace counseling and evaluation  C_g_: Individual guidance with home-based exercises and general information | Pain, work ability, function (VAS, DASH) | **Provider**  Physiotherapists  **Frequency**  3-4 times/week for exercises  **Duration**  60 minutes  **Follow-up**  6,12 months |
| Tsuboi et al [21] | Japan | C-  RCT | Baseline: IG=16, CG=13  Follow-up: IG=16, CG=13 | 38  I_g1_= 42.5  C_g_= 32 | Chronic low back pain | Office workers | I_g_: Workplace active rest programme (WARP) involving frequent stand-up and brief exercises during work, supported by an LBP workshop  C_g_: No intervention | Pain, functional disability (VAS, ODI) | **Provider**  Physiotherapists  **Frequency**  5 sessions per day  **Duration**  Few minutes per session  **Follow-up**  16 weeks |

**Key:** OA: Osteoarthritis, C-RCT: Cluster Randomised controlled trial, RCT: Randomised controlled trial, CMSDs: Chronic musculoskeletal disorders, Ig=Intervention group, Cg=Control group. Functional outcomes are used interchangeable. Sample sizes are reported separately for baseline and follow-up where available. Where group-specific follow-up data were not reported, totals or “not reported” are provided. Follow-ups refer to the timing of outcome assessment and not the duration of the intervention.

**Table 5: Results**

| **EXERCISE-BASED INTERVENTIONS** | | | | | |
| --- | --- | --- | --- | --- | --- |
| **Author, Sample Size, Overall quality** | **Results** | | | | |
| Alqhtani et al. 2023  N=60 | **Intervention (mean change ± SD)** | | **Control (mean change ± SD)** | | **Between-group difference (mean difference, 95% CI, or p-value)** |
|  | **Pain (VAS 0-10)** | | | | |
|  | **3 Weeks**  Δ=-2.14 (±1.15)  **6 Weeks**  Δ=-3.57 (±1.16) | | **3 Weeks**  Δ=-0.96 (±0.76)  **6 Weeks**  Δ=-2.46 (±0.90) | | Group × time interaction  p = 0.047*  *Standardised effect size not reported* |
| Alvani et al. 2021  N=30 | **Intervention (mean change ± SD)** | | **Control (mean change ± SD)** | | **Between-group difference (mean difference, 95% CI, or p-value)** |
|  | **Pain (VAS 0-10)** | | | | |
|  | **8 Weeks**  Δ = -2.06 ± 1.96 | | **8 Weeks**  Δ = +1.20 ± 1.29  The control group experienced an increase in pain intensity. | | **8 Weeks**  Group × time p = 0.001*  *Standardised effect size not reported* |
|  | **Functional disability (ODI 0-100)** | | | | |
|  | **8 Weeks**  Δ = -6.93 ± 8.68 | | **8 Weeks**  Δ = +3.33 ± 11.92  The control group experienced an increase in functional disability. | | **8 Weeks**  Group × time p = 0.044*  *Standardised effect size not reported* |
| Beltrán et al. 2024  N=44 | **Intervention (mean change ± SD)** | | **Control (mean change ± SD)** | | **Between-group comparison** |
|  | **Pain (NPRS 0-10)** | | | | |
|  | **8 Weeks**  Δ = −4.26 | | **8 Weeks**  Δ = −3.74 | | **8 Weeks**  MD = 0.19 (95% CI −2.64 to 2.26)  No significant difference  *Standardised effect size not reported* |
|  | **Shoulder Pain and Disability Index (SPADI 0-100)** | | | | |
|  | **8 Weeks**  Δ = -17.76 | | **8 Weeks**  Δ = -18.39 | | **8 Weeks**  MD = 1.23 (95% CI −13.54 to 16.00)  No significant difference  *Standardised effect size not reported* |
| Bernardelli et al. 2020  N=101 | **Intervention (mean change ± SD)** | | **Control (mean change ± SD)** | | **Between-group difference (mean difference, 95% CI, or p-value)** |
|  | **Functional Disability (RMDQ 0-24)** | | | | |
|  | **7 weeks**  Δ = -2.1 ± 3.3 | | **7 weeks**  Δ = -2.5 ± 4.3 | | **7 weeks**  p = 0.93  *Standardised effect size not reported* |
|  | **Functional disability (ODI 0-50)** | | | | |
|  | **7 weeks**  Δ = -3.5 ± 8.5 | | **7 weeks**  Δ = -0.3 ± 7.1 | | **7 weeks**  p = 0.02  *Standardised effect size not reported* |
| Ghavamabadi et al. 2022  N=250 | **Intervention (mean ± SD)** | | **Control (mean ± SD)** | | **Between-group difference (mean difference, 95% CI, or p-value)** |
|  | **Pain (VAS 0-10)** | | | | |
|  | **2 Months**  4.13 ± 1.81 | | **2 Months**  5.22 ± 2.31 | | **2 Months**  p < 0.001*  *Standardised effect size not reported* |
|  | **Functional Disability (RMDQ 0-24)** | | | | |
|  | **2 Months**  8.91 ± 6.51 | | **2 Months**  11.09 ± 5.22 | | **2 Months**  p < 0.001*  *Standardised effect size not reported* |
|  | **Quality of Life (SF-36)** | | | | |
|  | **2 Months**  **Physical Aspect:**  64.00 ± 12.01  **Psychological Aspect:**  69.00 ± 15.12 | | **2 Months**  **Physical Aspect:**  40.00 ± 14.13  **Psychological Aspect:**  49.50 ± 4.44 | | **2 Months**  p = 0.021  p = 0.034  *Standardised effect size not reported* |
| Jakobsen et al. 2018  N=200 | **Intervention (mean change ± SD)** | | **Control (mean change ± SD)** | | **Between-group difference (mean difference, 95% CI, or p-value)** |
|  | **Pain (0-10 scale)** | | | | |
|  | **10 Weeks**  Lower back: Δ = -0.9  Feet: Δ = -0.8  Upper back: Δ = -0.6 | | **10 Weeks**  Lower back: Δ = -0.2  Feet: Δ = -0.1  Upper back: Δ = -0.1 | | Lower back: MD = −0.7 (95% CI −1.1 to −0.3), p = 0.001  Feet: MD = −0.6 (95% CI −0.9 to −0.2), p = 0.002  Upper back: MD = −0.5 (95% CI −0.9 to −0.1), p = 0.009 |
|  | **Pressure Pain Threshold (kPa)** | | | | |
|  | **10 Weeks**  Lower back: Δ = +41  Tibialis anterior: Δ = +26 | | **10 Weeks**  Lower back: Δ = -46  Tibialis anterior: Δ = -52 | | Lower back: MD = +41 (95% CI 13 to 70), p = 0.005  Tibialis anterior: MD = +26 (95% CI 3 to 49), p = 0.029 |
| Kang et al. 2019  N=30 | **Intervention (mean change ± SD)** | | **Control (mean change ± SD)** | | **Between-group difference (mean difference, 95% CI, or p-value)** |
|  | **Hand grip strength (kg)** | | | | |
|  | **8 Weeks**  Δ = +3.52 ± 2.03 | | **8 Weeks**  Δ = +0.57 ± 0.62 | | **8 Weeks**  p = 0.015*  **Effect size** = 1.01 |
|  | **AUSCAN Pain (0-100)** | | | | |
|  | **8 Weeks**  Δ = -21.6 ± 8.3 | | **8 Weeks**  Δ = -7.85 ± 5.46 | | **8 Weeks**  p < 0.001*  **Effect size** = 1.8 |
|  | **AUSCAN Stiffness (0-100)** | | | | |
|  | **8 Weeks**  Δ = -16.8 ± 10.2 | | **8 Weeks**  Δ = -11.42 ± 7.18 | | **8 Weeks**  No significant difference |
|  | **AUSCAN Physical function (0-100)** | | | | |
|  | **8 Weeks**  Δ = -13.86 ± 4.54 | | **8 Weeks**  Δ = -10.28 ± 14.41 | | **8 Weeks**  p = 0.020  **Effect size =** 0.6 |
| Karatrantou et al. 2024  N=76 | **Intervention (mean change ± SD)** | | **Control (mean change ± SD)** | | **Between-group difference (mean difference, 95% CI, or p-value)** |
|  | **Pain (NMQ 0-10)** | | | | |
|  | **6 months**  Neck: Δ = -3.2 ± 1.1  Shoulders: Δ = -2.8 ± 1.0  Upper Back: Δ = -2.5 ± 1.1  Lower Back: Δ = -2.9 ± 1.2  Wrist/Hands: Δ = -2.1 ± 1.0  Legs: Δ = -2.6 ± 1.2 | | **6 months**  Neck: Δ = -0.2 ± 0.3  Shoulders: Δ = -0.3 ± 0.4  Upper Back: Δ = -0.1 ± 0.2  Lower Back: Δ = -0.4 ± 0.3  Wrist/Hands: Δ = -0.1 ± 0.2  Legs: Δ = -0.3 ± 0.3 | | **6 months**  p < 0.001* for all regions in favour of the intervention group  *Standardised effect size not reported* |
|  | **Strength (Dynamometer)** | | | | |
|  | **6 months**  **Handgrip Strength (kg)**  Right Hand: Δ = +4.0 ± 4.3  Left Hand: Δ = +4.5 ± 5.3  **Cervical Strength (lb)**  Forward Flexion: Δ = +5.3 ± 8.4,  Extension: Δ = +7.4 ± 11.5  **Leg Strength (kg)**  Δ = +22.7 ± 15.0  **Back Strength (kg)**  Δ = +19.7 ± 13.7 | | **6 months**  **Handgrip Strength (kg)**  Right Hand: Δ = -0.1 ± 3.5  Left Hand: Δ = 0.0 ± 2.6  **Cervical Strength (lb)**  Forward Flexion: Δ = -0.1 ± 5.6  Extension: Δ = +0.2 ± 3.2  **Leg Strength (kg)**  Δ = +0.6 ± 3.0  **Back Strength (kg)**  Δ = +0.5 ± 3.7 | | **6 months**  p < 0.001* for all regions in favour of the intervention group  *Standardised effect size not reported* |
| Korshøj et al. 2018  N=116 | **Intervention (mean change ± SD)** | | **Control (mean change ± SD)** | | **Between-group difference (mean difference, 95% CI, or p-value)** |
|  | **Pain (NMQ 0-10)** | | | | |
|  | **4 Months**  **Neck:** Δ = -1.15 ± 0.58  **Shoulders:** Δ = -1.38 ± 0.56  **Arms/Wrists:** Δ = -1.18 ± 0.55  **Upper Back:** Δ = -0.60 ± 0.60  **Lower Back:** Δ = -0.75 ± 0.56  **Hip:** Δ = -0.21 ± 0.38  **Knees:** Δ = +0.66 ± 0.45  **Feet/Ankles:** Δ = -0.72 ± 0.53  **12 Months**  **Neck:** Δ = -1.26 ± 0.5  **Shoulders:** Δ = -1.52 ± 0.44  **Arms/Wrists:** Δ = -1.30 ± 0.43  **Upper Back:** Δ = -0.72 ± 0.53  **Lower Back:** Δ = -0.82 ± 0.49  **Hip:** Δ = -0.16 ± 0.41  **Knees:** Δ = +0.81 ± 0.44  **Feet/Ankles:** Δ = -0.91 ± 0.47 | | **4-Months**  **Neck:** Δ = +0.42 ± 0.35  **Shoulders:** Δ = +0.63 ± 0.38  **Arms/Wrists:** Δ = +0.51 ± 0.40  **Upper Back:** Δ = +0.30 ± 0.34  **Lower Back:** Δ = +0.50 ± 0.43  **Hip:** Δ = -0.04 ± 0.27  **Knees:** Δ = +0.38 ± 0.31,  **Feet/Ankles:** Δ = -0.14 ± 0.19  **12 Months**  **Neck:** Δ = +0.53 ± 0.42  **Shoulders:** Δ = +0.66 ± 0.66  **Arms/Wrists:** Δ = +0.57 ± 0.57  **Upper Back:** Δ = +0.45 ± 0.45  **Lower Back:** Δ = +0.73 ± 0.73  **Hip:** Δ = -0.07 ± 0.07  **Knees:** Δ = +0.48 ± 0.48  **Feet/Ankles:** Δ = -0.09 ± 0.09 | | **4-Months**  Hip: MD = +0.61 (95% CI 0.11 to 1.12), p = 0.02 → worse in intervention  All other regions: no significant differences  **12 Months**  Neck: MD = −1.26 (95% CI −2.25 to −0.28), p = 0.01  Shoulders: MD = −1.52 (95% CI −2.39 to −0.65), p < 0.01  Arms/Wrists: MD = −1.30 (95% CI −2.15 to −0.44), p < 0.01*s*  Knees: MD = +0.81 (95% CI −0.07 to 1.69), p = 0.07 (trend towards worse)  Feet/Ankles: MD = −0.91 (95% CI −1.83 to 0.01), p = 0.05 (borderline)  Upper back, lower back, hip: no significant differences |
| Makki et al. 2024  N=70 | **Intervention (mean change ± SD)** | | **Control (mean change ± SD)** | | **Between-group difference (mean difference, 95% CI, or p-value)** |
|  | **Pain (NMQ 0-10)** | | | | |
|  | **4 weeks**  **Neck**  Δ = -2.00 ± 1.5  **Shoulder**  Δ = -1.00 ± 2.4  **Upper Back**  Δ = -1.00 ± 1.77  **Lower Back**  Δ = -2.50 ± 1.88 | | **4 weeks**  **Neck**  Δ = +1.00 ± 0.5  **Shoulder**  Δ≈0  **Upper Back**  Δ = +2.00 ± 2.00  **Lower Back**  Δ = +0.88 ± 2.88 | | **4 weeks**  **Neck**  p < 0.04*  **Shoulder**  p < 0.0001*  **Upper Back**  p < 0.0001*  **Lower Back**  p < 0.0001*  *Standardised effect size not reported* |
| Moreira et al. 2021  N=90 | **Intervention (mean change ± SD)** | | **Control (mean change ± SD)** | | **Between-group difference (mean difference, 95% CI, or p-value)** |
|  | **Strength (kgf)** | | | | |
|  | **12 Weeks**  **Trunk Flexors Strength**  Δ= +4.59 ± 6.01  **Back Extensors Strength (kgf)**  Δ= +0.16 ± 13.03 | | **12 Weeks**  **Trunk Flexors Strength**  Δ=-1.21 ± 4.82  **Back Extensors Strength (kgf)**  Δ= +0.03 ± 18.38 | | **12 Weeks**  **Trunk Flexors Strength**  p = 0.002 *  **Effect size d=** 0.77  **Back Extensors Strength**  No significant difference p = 0.307  **Effect size d=** 0.16 |
|  | **Pressure Pain Threshold (PPT kgf/cm²)** | | | | |
|  | **12 Weeks**  **Right Dorsal Longissimus**  Δ= +1.61 ± 2.03  **Left Dorsal Longissimus**  Δ= +1.66 ± 2.13 | | **12 Weeks**  **Right Dorsal Longissimus**  Δ= -0.5 ± 2.34  **Left Dorsal Longissimus**  Δ= -0.67 ± 2.49 | | **12 Weeks**  **Right Dorsal Longissimus**  p = 0.001 *  **Effect size d=** 0.82  **Left Dorsal Longissimus**  p = 0.001*  **Effect size d=** 0.89 |
| **Low back symptoms (clinical exam)** | | | | | |
|  | N/A | | N/A | | p = 0.002 (in favour of intervention)*  OR = 6.25 (95% CI 1.6–24.1) |
| Passos et al. 2024  N=63 | **Intervention (mean change ± SD)** | | **Control (mean change ± SD)** | | **Between-group difference (mean difference, 95% CI, or p-value)** |
|  | **Pain (0-10)** | | | | |
|  | **8 Weeks**  Δ = -4.55 ± 3.57 | | **8 Weeks**  Δ = -3.81 ± 2.85 | | **8 Weeks**  MD = 0.43 (95% CI −2.11 to 2.96), p > 0.05  *Standardised effect size not reported* |
|  | **Disability (Roland-Morris 0-24)** | | | | |
|  | **8 Weeks**  Δ = -4.45 ± 5.31 | | **8 Weeks**  Δ = -4.43 ± 3.05 | | **8 Weeks**  MD = −1.53 (95% CI −1.85 to 4.92), p > 0.05  *Standardised effect size not reported* |
| Phattharasupharerk et al. 2019  N=72 | **Intervention (mean change ± SD)** | | **Control (mean change ± SD)** | | **Between-group difference (mean difference, 95% CI, or p-value)** |
|  | **Pain (VAS 0-100)** | | | | |
|  | **6 Weeks**  Δ = -35.7 ± 20.5 | | **6 Weeks**  Δ = -1.3 ± 20.9 | | **6 Weeks**  p < 0.001 *  *Standardised effect size not reported* |
|  | **Physical impairment (RMDQ 0-24)** | | | | |
|  | **6 Weeks**  Δ = -2.6 ± 3.6 | | **6 Weeks**  Δ = 0.4 ± 3.2 | | **6 Weeks**  p = 0.022 *  *Standardised effect size not reported* |
| **MULTICOMPONENT INTERVENTIONS** | | | | | |
| Rantonen et al. 2018  N=126 | **Intervention (mean change ± SD) 1** | **Intervention (mean change ± SD) 2** | **Intervention (mean change ± SD) 3** | **Control (mean change ± SD)** | **Between-group difference (mean difference, 95% CI, or p-value)** |
|  | **Physical impairment (RMDQ 0-24)** | | | | |
|  | Not reported | Not reported | Not reported | Not reported | **24 Months**  MD = −3 (95% CI −5 to −1),  p < 0.001* in favour of the intervention group 1  **Effect size d**= 0.7  MD = −3 (95% CI −5 to −1), in favour of the intervention group 2  **Effect size d**= 0.7  Not significant results for the intervention group 3 |
|  | **Pain (VAS, 0-100 mm)** | | | | |
|  | Not reported | Not reported | Not reported | Not reported | **24 Months**  MD = −6 (95% CI −11 to −1), p < 0.05  **Effect size d**= 0.6  p < 0.05* in favour of the intervention group 2  **Effect size d**= 0.6  Not significant results for the intervention group 3 |
|  | **Quality of Life (EQ-5D, 0-1)** | | | | |
|  | Not reported | Not reported | Not reported | Not reported | **24 Months**  MD = 0.06 (95% CI 0.01 to 0.10), p < 0.01* in favour of the intervention group 1  **Effect size d**= 0.4  MD = 0.05 (95% CI 0.00 to 0.10), p < 0.05* in favour of the intervention group 2  Not significant results for the intervention group 3 |
| Shariat et al. 2018  N=180 | **Intervention (mean change ± SD) 1** | **Intervention  group 2** | **Intervention  group 3** | **Control (mean change ± SD)** | **Between-group difference (mean difference, 95% CI, or p-value)** |
|  | **Musculoskeletal Disorders Questionnaire (CMDQ)** | | | | |
|  | **6 Months**  **Neck Pain**  Δ = -1.9 ± 1.2  **Shoulder Pain**  Δ = -2.0 ± 1.3  **Lower Back Pain**  Δ = -3.2 ± 1.8 | **6 Months**  **Neck Pain**  Δ = -2.0 ± 1.3*  **Shoulder Pain**  Δ = -2.1 ± 1.4  **Lower Back Pain**  Δ = -3.3 ± 1.9 | **6 Months**  **Neck Pain**  Δ = -2.4 ± 1.4  **Shoulder Pain**  Δ = -2.5 ± 1.5  **Lower Back Pain**  Δ = -3.6 ± 2.0 | **6 Months**  **Neck Pain**  Δ = -0.3 ± 0.5  **Shoulder Pain**  Δ = -0.5 ± 0.6  **Lower Back Pain**  Δ = -0.7 ± 0.8 | **6 Months**  **Neck Pain**  Exercise vs control:  MD = −10.55 (95% CI −14.36 to −6.74), p < 0.001*  Combined (exercise + ergonomic) vs control:  MD = −9.99 (95% CI −13.63 to −6.36), p < 0.001*  Ergonomic only:  No significant difference at 6 months  **Shoulder Pain**  Shoulder pain  Exercise vs control:  MD = −12.17 (95% CI −16.87 to −7.47), p < 0.001*  Combined vs control:  MD = −11.12 (95% CI −15.59 to −6.65), p < 0.001*  Ergonomic only:  No significant difference at 6 months  **Lower Back Pain**  Exercise vs control:  MD = −7.8 (95% CI −11.08 to −4.53), p < 0.001*  Combined vs control:  MD = −6.87 (95% CI −10 to −3.74), p < 0.001*  Ergonomic only:  No significant difference at 6 months |
| Ting et al. 2019  N=97 | **Intervention (mean change ± SD) 1** | | **Intervention (mean change ± SD) 2** | | **Between-group difference (mean difference, 95% CI, or p-value)** |
|  | **Work Ability** | | | | |
|  | **12 weeks**  Δ =8.82 ± 0.95  **12 months**  Δ =8.93 ±1.14 | | **Not provided** | | **12 weeks**  *No significant difference*  **12 months**  *No significant difference* |
| Trøstrup et al. 2023  N=109 | **Intervention (Mean score at follow-up ± SD)** | | **Control (Mean score at follow-up ± SD)** | | **Between-group difference (mean difference, 95% CI, or p-value)** |
|  | **Oxford Shoulder Score (OSS)** | | | | |
|  | **6 months**  Δ =40.4 ± 5.5  **12 months**  Δ =40.3 ± 7.3 | | **6 months**  Δ =40.1 ± 5.7  **12 months**  Δ =40.4 ± 5 | | **6 months**  MD = 0.3 (95% CI −1.6 to 2.2), No significant difference  **12 months**  No significant difference |
|  | **Pain (NRS 0-10)** | | | | |
|  | **6 months**  Δ =1.9 ± 1.9  **12 months**  Δ =1.7 ± 2.0 | | **6 months**  Δ =2.0 ± 1.8  **12 months**  Δ =2.4 ± 2.1 | | **6 months**  MD = 0.1 (95% CI −0.6 to 0.8), No significant difference  **12 months**  No significant difference |
| Imai et al. 2021  N=104 | **Intervention (mean change ± SD)** | | **Control (mean change ± SD)** | | **Between-group difference (mean difference, 95% CI, or p-value)** |
|  | **Absolute Presenteeism (WHO-HPQ)** | | | | |
|  | **6 months**  Δ = +6.9 ± 13.4 | | **6 months**  Δ = -4.2 ± 20.1 | | **6 months**  Group × time p = 0.001*  (η² = 0.94) |
|  | **Relative Presenteeism (WHO-HPQ)** | | | | |
|  | **6 months**  Δ = +0.14 ± 0.48 | | **6 months**  Δ = +0.14 ± 0.48 | | **6 months**  Δ = +0.14 ± 0.48 |
|  | **Pain (NRS 0-10)** | | | | |
|  | **6 months**  Δ = -1.6 ± 1.9 | | **6 months**  Δ = -1.6 ± 1.9 | | **6 months**  Δ = -1.6 ± 1.9 |
|  | **Widespread Pain Index (WPI)** | | | | |
|  | **6 months**  Δ = -1.1 ± 2.0 | | **6 months**  Δ = -1.1 ± 2.0 | | **6 months**  Δ = -1.1 ± 2.0 |
|  | **Quality of life (EQ-5D)** | | | | |
|  | **6 months**  Δ = +0.09 ± 0.13 | | **6 months**  Δ = +0.09 ± 0.13 | | **6 months**  Δ = +0.09 ± 0.13 |
| Hergenroeder et al. 2022  N=27 | **Intervention (mean change ± SD)** | | **Control (mean change ± SD)** | | **Between-group difference (mean difference, 95% CI, or p-value)** |
|  | **Pain (SF-36 Pain Subscale)** | | | | |
|  | **6 months**  Not reported | | **6 months**  Not reported | | **6 months**  Not reported |
|  | **Productivity (HWQ Subscale)** | | | | |
|  | **6 months**  Not reported | | **6 months**  Not reported | | **6 months**  Not reported |
|  | **Presenteeism (SPS-6 Subscale)** | | | | |
|  | **6 months**  Not reported | | **6 months**  Not reported | | **6 months**  Not reported |
| Alshehre et al. 2023  N=60 | **Intervention (mean change ± SD)** | | **Control (mean change ± SD)** | | **Between-group difference (mean difference, 95% CI, or p-value)** |
|  | **Pain (NPRS 0-10)** | | | | |
|  | **4 Weeks**  Δ = -1.6 ± 0.5  **8 Weeks**  Δ = -3.1 ± 0.5, | | **4 Weeks**  Δ = -1.6 ± 0.5  **8 Weeks**  Δ = -3.1 ± 0.5, | | **4 Weeks**  Δ = -1.6 ± 0.5  **8 Weeks**  Δ = -3.1 ± 0.5, |
|  | **Neck Disability Index (NDI)** | | | | |
|  | **4 Weeks**  Δ = -5.98 ± 7.7  **8 Weeks**  Δ = -15.33 ± 7.7 | | **4 Weeks**  Δ = -5.98 ± 7.7  **8 Weeks**  Δ = -15.33 ± 7.7 | | **4 Weeks**  Δ = -5.98 ± 7.7  **8 Weeks**  Δ = -15.33 ± 7.7 |
|  | **Quality of Life (SF-36)** | | | | |
|  | **4 Weeks**  Δ = +4.4 ± 6.5  **8 Weeks**  Δ = +12.6 ± 7.5 | | **4 Weeks**  Δ = +4.4 ± 6.5  **8 Weeks**  Δ = +12.6 ± 7.5 | | **4 Weeks**  Δ = +4.4 ± 6.5  **8 Weeks**  Δ = +12.6 ± 7.5 |
| Tsuboi et al. 2021  N=29 | **6 months**  Δ = +6.9 ± 13.4 | | **6 months**  Δ = -4.2 ± 20.1 | | **6 months**  Group × time p = 0.001*  (η² = 0.94) |
|  | **Relative Presenteeism (WHO-HPQ)** | | | | |
|  | **6 months**  Δ = +0.14 ± 0.48 | | **6 months**  Δ = -0.01 ± 0.47 | | **6 months**  Group × time p = 0.04  (η² = 0.04) |
|  | **Pain (NRS 0-10)** | | | | |
|  | **6 months**  Δ = -1.6 ± 1.9 | | **6 months**  Δ = 0.0 ± 2.6 | | **6 months**  Group × time p = 0.001  (η² = 0.10) |
|  | **Widespread Pain Index (WPI)** | | | | |
|  | **6 months**  Δ = -1.1 ± 2.0 | | **6 months**  Δ = -0.1 ± 2.1 | | **6 months**  Group × time p = 0.03  (η² = 0.046) |
| **ERGONOMIC INTERVENTIONS** | | | | | |
| Lee et al. 2021  N=95 | **Intervention (mean change ± SD)** | | **Control (mean change ± SD)** | | **Between-group difference (mean difference, 95% CI, or p-value)** |
|  | **Quality of life (EQ-5D)** | | | | |
|  | **6 months**  Δ = +0.09 ± 0.13 | | **6 months**  Δ = +0.02 ± 0.14 | | **6 months**  Group × time p = 0.051  (η² = 0.05) |
|  | **Pain (NPRS 0-10)** | | | | |
|  | **12 weeks**  Neck: Δ = -1.2 ± 0.3  Shoulder: Δ = -1.9 ± 1.9  Upper Back: Δ = -1.6 ± 0.3  Wrist/Hand: Δ = -1.1 ± 0.3  Lower Back: Δ = -1.2 ± 0.5  Elbow: Δ = +0.2 ± 0.3  Hip/Thigh: Δ = -0.4± 0.3  Knee: Δ = +0.1± 0.3  Foot/Ankle: Δ = -0.1± 0.2  **24 weeks**  Neck: Δ = -0.7 ± 0.7  Shoulder: Δ = -0.7 ± 0.4  Upper Back: Δ = -1 ± 0.4  Wrist/Hand: Δ = -1.3 ± 0.2  Lower Back: Δ = -1.2 ± 0.4  Elbow: Δ = +0.2 ± 0.2  Hip/Thigh: Δ = -0.6± 0.2  Knee: Δ = +0.3± 0.2  Foot/Ankle: Δ = +0.4± 0.2  **36 weeks**  Neck: Δ = -0.6 ± 0.2  Shoulder: Δ = -1.4 ± 0.5  Upper Back: Δ = -1.9 ± 0.3  Wrist/Hand: Δ = -1.2 ± 0.2  Lower Back: Δ = -1.8 ± 0.3  Elbow: Δ = +0.1 ± 0.2  Hip/Thigh: Δ = -0.7± 0.2  Knee: Δ = +0.1± 0.2  Foot/Ankle: Δ = +0.4± 0.2 | | **12 weeks**  **Neck:** Δ = +0.6 ± 0.4  **Shoulder:** Δ = +0.5 ± 0.4  **Upper Back:** Δ = +0.9 ± 0.4  **Wrist/Hand:** Δ =-1.0 ± 0.4  **Lower Back:** Δ = +0.1 ± 0.4  **Elbow:** Δ = -0.3± 0.4  **Hip/Thigh:** Δ = +0.2± 0.4  **Knee:** Δ = +0.6± 0.4  **Foot/Ankle:** Δ = +0.1± 0.2  **24 weeks**  **Neck:** Δ = +1.8 ± 0.5  **Shoulder:** Δ = +2 ± 0.5  **Upper Back:** Δ = +1.5 ± 0.5  **Wrist/Hand:** Δ = +0.4 ± 0.5  **Lower Back:** Δ = -1.2± 0.5  **Elbow:** Δ = -0.4± 0.5  **Hip/Thigh:** Δ = +0.6± 0.5  **Knee:** Δ = +0.6± 0.5  **Foot/Ankle:** Δ = +0.0± 0.0  **36 weeks**  **Neck:** Δ = +0.6 ± 0.3  **Shoulder:** Δ = +0.2 ± 0.3  **Upper Back:** Δ = +0.2 ± 0.3  **Wrist/Hand:** Δ = +0.9 ± 0.3  **Lower Back:** Δ = -1.8± 0.3  **Elbow:** Δ = +0.2± 0.3  **Hip/Thigh:** Δ = +0.8± 0.3  **Knee:** Δ = +1.1± 0.3  **Foot/Ankle:** Δ = -0.6± 0.3 | | **Overall comparison**  *No significant group × time interaction (p > 0.05)*  Significant between-group differences found for: Neck, Shoulder, Upper back, Wrist/hand  p < 0.05 (in favour of intervention group) |
| Yaghoubitajani et al. 2022  N=36 | **Intervention (mean change ± SD) 1** | **Intervention (mean change ± SD) 2** | **Control (mean change ± SD)** | | **Between-group difference (mean difference, 95% CI, or p-value)** |
|  | **Neck-Shoulder Pain (NSP)** | | | | |
|  | **8 Weeks**  Δ=-3.92 | **8 Weeks**  Δ= -2.50 | **8 Weeks**  Δ=0.45 | | p = 0.007*; Effect size (η²) = 0.306 (ANCOVA, favouring online-supervised group) |
|  | **Sick Leave** | | | | |
|  | **8 Weeks**  Δ=0.17 | **8 Weeks**  Δ=3.63 | **8 Weeks**  Δ=0.09 | | No significant between-group differences (p ≥ 0.05) |
|  | **Physical Workability** | | | | |
|  | **8 Weeks**  Δ=0.17 | **8 Weeks**  Δ=0.50 | **8 Weeks**  Δ=0.27 | | p = 0.048*; Effect size (η²) = 0.055 (significant difference favouring online-supervised group) |
| **Notes:** Values are presented as mean change from baseline ± standard deviation (SD), unless otherwise stated. Where available, between-group mean differences and 95% confidence intervals are reported. Where these were not reported in the original studies, p-values are presented and group-level data are shown. Where repeated-measures analyses were used, between-group differences are reported as group × time interaction p-values, representing overall differences between groups across all timepoints. Where reported, Δ represents within-group mean change from baseline to follow-up. P-values indicate the statistical significance of the observed changes. Standard deviations (SD) for the mean differences are sometimes provided explicitly or implied through confidence intervals. Beta coefficient (b) represents the effect size or the degree of change in the outcome variable. All analyses are performed using the intent-to-treat principle. β represents regression coefficient. *Statistically significant at p < 0.05, p<0.01 and p<000.1. The colourful indication represents the overall score of RoB 2 using the risk-of-bias assessments with “traffic lights” (Figure 1). Abbreviations: MD = mean difference between groups, Roland-Morris Disability Questionnaire (RDQ), Work Limitations Questionnaire (WLQ), Oswestry Disability Index (ODI), Widespread Pain Index (WPI), EuroQol 5 Dimensions 5 Levels (EQ5D-5L), Brief Pain Inventory (BPI), Nordic Musculoskeletal Questionnaire (NMQ), Numerical Pain Rating Scale (NPRS), Australian/Canadian Osteoarthritis Hand Index (AUSCAN), Pressure Pain Threshold (PPT), World Health Organization Health and Work Performance Questionnaire (WHO-HPQ), Roland-Morris Disability Questionnaire (RMDQ), Short Form Health Survey-36 (SF-36), Health and Work Questionnaire (HWQ), Stanford Presenteeism Scale (SPS-6). | | | | | |
