# Supplementary material for: Workplace interventions for chronic musculoskeletal disorders: a systematic review
Source: BMJ Open. 2026 Jun 22;16(6):e115276. doi: 10.1136/bmjopen-2025-115276 (PMC13288689; doi:10.1136/bmjopen-2025-115276)
Supplement: Supplementary file 3 [file bmjopen-2025-115276.reviewer_comments.pdf]

PEER REVIEW HISTORY

BMJ Open publishes all reviews undertaken for accepted manuscripts. Reviewers are asked to complete a checklist review form and are provided with free text boxes to elaborate on their assessment. These free text comments are reproduced below.

ARTICLE DETAILS

Title (Provisional)

Workplace Interventions for Chronic Musculoskeletal Disorders: A Systematic Review

Authors

Skamagki, Glykeria; Greaves, Colin J; Shrestha, Ruby; Stathi, Afroditi

---

VERSION 1 - REVIEW

Reviewer 1

Name Liebers, Falk

Affiliation Federal Institute for Occupational Safety and Health (BAuA), Nöldnerstr. 40-42, 10317 Berlin, Germany, FB 3

Date 17-Feb-2026

COI None

Peer-Review for BMJ Open

Date of the review

• 17.02.2026

Title of the manuscript

• "Workplace Interventions for Chronic Musculoskeletal Disorders: A Systematic Review"

Manuscript Identifier:

• ID = bmjopen-2025-115276 - Original research

• First draft

Authors of the draft:

• Not blinded to the reviewer: G. Skamagki et al.

Type of the manuscript

• Systematic review

Reviewer's report

Generally

Thank you for the opportunity to review the excellent manuscript.

The objective of the study is clearly given. The systematic review evaluated the effectiveness of workplace interventions for CMSDs, identified key intervention components, and assessed their practical application for improving health and work-related outcomes.

The main aspects to give reasons for the systematic reviews is provided. Scientific background, the aim of the study, study design (systematic review), search and selection of the studies, methodological approach for study assessment and data extraction, definition for the intervention of interest, the outcomes of interest, the approach for summarizing the studies, main results, and a discussion are reported. The systematic review is following the standards of reporting.

The originality is high. It adds evidence regarding the effect of workplace interventions in the case of chronic musculoskeletal disorders. The information is important for practitioners in the workplace setting. The evidence presented is scientifically and reliable. The research question is it clearly defined and appropriately answered.

Study design is the reported and adequate. The selection of studies relevant for the topic is adequately described and conditions are well defined. The used methods of the systematic review are adequately described and up to date. The results are credible and well-presented. Interpretation and conclusions are they warranted by and sufficiently derived from/focused on the data. The message is clear. References are they up to date and relevant. The Abstract reflects accurately what the paper says. The paper complies with reporting standards (Prisma). The Prisma 2020 checklist for reporting of systematic reviews is included and is appropriate.

Generally, the manuscript is very interesting, clear and understandable written. The length of the manuscript is large, but is necessary to report the results of the systematic review. Shortening of the text would reduce the quality.

The reviewer has not to provide any major remarks for revision or major considerations. There are only minor edits (see below). I would like provide some recommendation regarding the structure of reporting to increase the clarity of the manuscript (see below, section "methods" and "results"). The manuscript is within the scope of BMJ Open. The reviewer recommends the manuscript for publication after minor revision.

• Comment #01 (Table 3 and Figure 2; page 7 and 8; recommendation): Table 3 and figure 2 include nearly the same information or statement. All included studies are listed. The risk of bias is judged for 5 dimensions. Figure 2 visualize the information using colors. My recommendation is to delete Figure 2 or Table 3 to reduce the size of the manuscript.

• Comment #02 (Section "2.5 Risk of Bias Assessment"; page 6, lines 183 ff.; recommendation): The last sentence of the paragraph does not only describe the methodological approach, but refers to table 3 and figure 2 and therefore includes first

results. The selected list of studies is a result of the systematic review. This is reported in section 3 “Results” and in section 3.3 “Quality appraisal”. It is not a usual approach to present the list of selected studies in section methods. My recommendation is, to delete the link to table 3 and figure 2 in section 2.5: “… The results of this assessment [ROB assessment] are presented in a detailed table (or figure). A traffic light system is employed visually depict the overall risk of bias for each study, providing an intuitive understanding of the quality of evidence across the studies.” The link to table 3 and figure 2 is then given in section 3.

• Comment #03 (Section 3.3 Quality appraisal; edit): Please correct the wrong link to table 4 in section 3.3. I think, “Table 3” is correct. Corrected sentence “A critical appraisal of the 23 studies included in this systematic review was conducted to assess the quality and reliability of the evidence presented (Table 3, Figure 2).”?

• Comment #04 (Section “3 Results”, subsection “3.5. Summary of Effectiveness Data”; recommendation): The results of all selected studies are described in the sections 3.5.1 to 3.5.4. The authors differentiate four types of the report of interventions. Unfortunately, table 5 “Study characteristics” and table 6 “Results and Outcomes” are sorted in alphabetic order by the authors of the selected studies, not subdivided by the for types of report of interventions. It is very complicated for the reader to get an overview reading sections 3.5.1 to 3.5.4 and looking for details of the single studies in tables 5 and 6. My recommendation is: Define the categories of intervention like “Different Physical Exercise Interventions”, “Usual care of Ergonomics”, “Digital and alternative Intervention” and other / traditional approaches early (section 3.2) and subdivide the list of studies in Table 4 to 6 consequently by these types of interventions.

• Comment #04 (Section 3.1 “Study characteristics”, page 9, lines 27 ff; recommendation): Tables 5 and 6 are reporting the characteristics of the studies in detail. Very impressive. But the tables are very long. Consider to provide both tables or one of the both as additional material to reduce the size of the manuscript.

**Reviewer**	**2**
**Name**	**Kjeken, Ingvild**
**Affiliation**	**Diakonhjemmet Sykehus**
**Date**	**18-Feb-2026**
**COI**	**None**

Thank you for the opportunity to review the manuscript “Workplace Interventions for Chronic Musculoskeletal Disorders: A Systematic Review.” The manuscript presents a review of randomized controlled trials evaluating the effects of various workplace interventions.

As the authors note, updated evidence on which workplace interventions can effectively support workers in reducing absenteeism and presenteeism is indeed important.

However, there are several issues within the manuscript that require attention. Please find my comments below.

Major comments:

2.3.1. Inclusion criteria: I suggest adding RCT as an inclusion criterion.

2.5. Risk-of-bias assessment: I suggest removing Table 3, as the same information is already presented in Figure 2 in a format that is much easier for readers to interpret.

3.1. Study characteristics – Table 5: Regarding the reporting of sample sizes, I would like to see the number of participants in each group at baseline as well as at each follow up time point. As the table is currently presented, the reporting appears inconsistent. For example, in Beltrán et al. (2024), the number of participants per group is provided at baseline, whereas in the following study (Bernardelli et al. 2020), the numbers reported correspond to the follow up time point instead. Please ensure consistent reporting across studies.

I would also like to know which outcomes were assessed in each study, as well as the instruments used to measure them.

Further, I find the use of the term “length” (in the last column of the table) somewhat imprecise, as it could be interpreted as referring to the duration of the intervention. However, it appears that the term is being used to indicate the timing of the follow up assessment. I recommend rephrasing this to avoid ambiguity.

3.2. Types of interventions: The authors classify digital and software based interventions as a separate intervention type. I suggest distinguishing between the content of an intervention and its mode of delivery. For example, the content may consist of exercises, while the delivery mode may be face to face (individual or group based) or digital.

3.4. Outcome measures: It would be helpful to know how many of the included trials assessed outcomes such as pain, function, and absenteeism. The same applies to the summary of interventions: how many trials evaluated the effects of each intervention type?

3.5 Summary of effectiveness data: Regarding the discussion section, much of it consists of referring to the results of individual studies. I understand that this is a review with a narrative synthesis of findings, but I would still expect the authors to provide a more overarching summary. Readers can look up the details of the individual studies in the various tables themselves. For example, in the summary of the effects of physical exercise interventions, I would have liked to see a synthesis that conveys what the overall body of evidence suggests about this type of intervention in relation to different outcome measures.

4. Discussion: In general, I also the discussion somewhat disjointed, jumping back and forth between the content of the interventions, the outcomes, and the mode of delivery. This makes it difficult to follow.

Further, somewhat surprisingly, very few studies appear to use presenteeism and/or absenteeism as outcome measures, even though these are, in my view, the outcomes most relevant to workplace interventions. I suggest that this point be addressed in the discussion section.

Minor comments:

Table 1. PICO approach, Comparison: The phrase “any or none” may be interpreted as suggesting that some studies did not include a control group. Since all included studies were randomized controlled trials, please rephrase this section to make it explicit that a control group was always present due to the RCT design.

In table 6, please describe what kind of outcome the results are for (i.e. pain) with instrument in brackets, and not the instrument only (i.e. Neck Disability Index).

Please also take a look at the use of prepositions. One example is on page 9, line 51: ‘Definitions and details can be found on Table 4,’ where I believe the correct phrasing is ‘in Table 4.’

VERSION 1 - AUTHOR RESPONSE

REVIEWER 1	
**Comments**	**Response**
Table 3 and Figure 2 duplicate info	We agree and have removed Table 3 to reduce redundancy. The risk of bias is now presented only in Figure 2 (page 13), which provides a clearer visual summary.
Methods section incorrectly includes results	Thank you. The Risk of Bias section has been revised to remove references to results and tables. Descriptions of findings are now reported only in the Results section (page 13-17).
Table 4 should be Table 3	We thank the reviewer. All table numbering has been corrected throughout the manuscript. In particular, references previously made to Table 4 in section 3.3 have been corrected to Table 3.
Tables not aligned with intervention categories	Thank you for the comment. We have restructured the Results section and tables to align with predefined intervention categories (exercise, ergonomic, and multicomponent interventions), as described in section “Types of interventions” (pages 10-11) and reflected in Table 3 and supplementary materials.
Tables are too long	To improve readability and reduce the length of the manuscript, Table 5 has been moved to Supplementary Material 3. Key summary data remain in the main manuscript (Table 3),

	while detailed study characteristics are now presented in the supplementary files.
REVIEWER 2	
**Comments**	**Response**
Add RCT as inclusion criterion	Thank you. This has been explicitly added to the inclusion criteria (page 6), clarifying that only RCT and cluster-RCT studies were included.
Remove Table 3	Agreed. Original table 3 has been removed.
Sample sizes inconsistent	We have standardised reporting across studies, including baseline and follow-up sample sizes for each group where available.
Outcomes + measurement tools unclear	We thank the reviewer. A new column has been added to Table 3 specifying outcomes and corresponding measurement instruments.
"Length" unclear	We thank the reviewer. The term "length" has been replaced with "Follow-up" in Table 3 to improve clarity.
Content vs delivery mixed	Thank you. We have revised the classification of interventions to distinguish between intervention content (exercise, ergonomic, multicomponent) and mode of delivery (face-to-face, digital), as described in the Methods (page 10) and reflected in the Results.
Need counts of outcomes/interventions	Thank you. We have added a summary of the number of studies assessing key outcomes by intervention type (page 11)
Discussion too study-by-study	We thank the reviewer. The Discussion section has been fully revised to provide a more analytical synthesis of findings, focusing on overall patterns of effectiveness across intervention types rather than individual study descriptions (pages 25-30)
